# WNK1 Kinase Activity Is Required for the Functional Maintenance of Podocyte Structure

**DOI:** 10.1096/fj.202503839R

**Published:** 2026-02-11

**Authors:** Zhenan Liu, Eunyoung Lee, Shumeng Jiang, Joonho Yoon, Fouzia Ahmed, Mohammad A. Rahman, Philip Bleicher, Hani Y. Suleiman, Leslie A. Bruggeman, R. Tyler Miller, Audrey N. Chang

**Affiliations:** ^1^ Department of Internal Medicine University of Texas Southwestern Medical Center Dallas Texas USA; ^2^ National Heart, Lung, and Blood Institute National Institutes of Health Bethesda Maryland USA; ^3^ Department of Inflammation & Immunity, Lerner Research Institute Cleveland Clinic Cleveland Ohio USA; ^4^ Medicine Service VA North Texas Health Care System Dallas Texas USA; ^5^ Pak Center for Mineral Metabolism and Clinical Research UTSW Medical Center Dallas Texas USA

**Keywords:** actomyosin, Alport syndrome, cytoskeleton, NMII, podocytes, vinculin, WNK1

## Abstract

The filtration function of glomeruli requires slit diaphragms formed by interdigitating podocyte foot processes, which are actin‐based membrane protrusions. Failure in the maintenance of these cytoskeletal structures leads to foot process effacement, proteinuria, and progression to chronic kidney disease. We report WNK1 kinase activity is required for normal glomerular function *in vivo* and test the hypothesis that WNK1 kinase activity affects the structure of podocyte foot processes through modulation of actomyosin activity and focal adhesion complexes. Perturbation of cytoskeletal structure and focal adhesion signalosomes with WNK1 kinase inhibition supports a role for WNK1 in the maintenance of podocyte foot processes and sarcomere‐like structures (SLSs) that are induced in models of podocyte injury. Applicability of WNK1 kinase activity modulation toward treatment of podocyte injury was assessed using primary and immortalized podocyte cell lines developed from control and *Col4a3* ^*−/−*^ Alport Syndrome model mice. Collectively, the results provide compelling evidence that WNK1 kinase signalosome activity, which includes regulation of nascent focal adhesion formation and NMII activity at membrane protrusions and extensions, is necessary for physiological maintenance of slit diaphragms.

## Graphical abstract

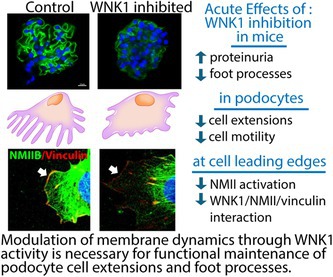

The acute effects of WNK1 kinase inhibition on podocyte structure and function were determined at the level of: 1) renal function, by measurements of urinary protein and imaging of glomerular synaptopodin, 2) podocyte cell, by measurements and localization of actomyosin proteins and their regulators, and 3) focal adhesions at cell membrane extensions. WNK1 kinase activity modulates actomyosin activation and focal adhesion interaction at membrane protrusions, supporting a role for WNK1 kinase activity in the maintenance of podocyte foot process structure.

## 1 Introduction

Disrupted podocyte actin cytoskeletal structure due to mutations in structural or regulatory proteins or their dysfunction is the foundation of many glomerular diseases and disease models. Various in vitro models of podocyte injury demonstrate reduced capacity to spread and generate traction force [1], indicating baseline dysfunction in focal adhesion formation and/or actomyosin regulation. Extensive interdigitation of cell processes among neighboring podocytes forms the slit diaphragm, essential components of the cellular sieve that retain proteins in the blood while allowing filtrate to enter the urine for reabsorption or excretion [2]. A characteristic of kidney disease progression is loss of podocyte structural complexity and adhesion strength with resultant proteinuria, filtration failure, and podocyte loss [3, 4].

Podocytes undergo hypertrophy and remodeling in response to neighboring podocyte loss or glomerular growth [5, 6]. The maintenance of arborized podocyte structures requires coordinated formation and activation of focal adhesions for dynamic regulation of the cell cytoskeleton that are responsive to hemodynamic forces and matrix stiffness [7, 8]. In order to retain glomerular filtration function while under pulsatile hemodynamic forces in a 3D environment, constant dynamic regulation of myosin activity at the membrane edges and in cell extensions is necessary for the formation and maintenance of the slit diaphragm and in focal adhesions for adhesion to the basement membrane. In the 2D environment, podocytes in culture migrate as they grow and divide, relying on assembly of nascent focal adhesions at leading edges of lamellipodial extensions, which undergo dynamic maturation to focal complexes and focal adhesions [9].

While the structures of actin filaments and focal adhesions at lamellipodia extensions and stress fibers of the cell are well documented in various cell types including podocytes [10], mechanisms that underlie regulation of membrane dynamics have not been defined clearly. Extending our previous measurements of WNK1 kinase activity in podocyte membrane blebs [11], we sought to determine whether WNK1 activity‐dependent regulation of membrane dynamics is physiologically relevant in vivo, and whether effects on podocyte cell structure were mediated through WNK1‐associated focal adhesion components that contribute to actomyosin regulation at lamellipodial extensions.

The collective results herein provide evidence that WNK1 kinase activity is necessary for the maintenance of podocyte slit diaphragm structure in vivo and that its activity contributes to mechanosensation in podocytes through formation of activity‐dependent signalosomes that modulates NMII activity at nascent focal adhesions and at cytoskeletal “sarcomere‐like structures” in pathological states. Additionally, rescue of the *Col4a3* ^*−/−*^ Alport Syndrome model glomerular structure with acute WNK1 activation supports WNK1 activity regulation as a potential mechanism of action for calcineurin inhibitors' beneficial effects in glomerular disease and suggests further study of the effects of WNK1 kinase activity on podocytes and glomerular capillaries may lead to identification of more specific targets.

## 2 Methods

*Isolation of Mouse Glomeruli‐* Glomeruli were isolated from minced tissue as previously detailed [11]. Glomeruli were maintained in DMEM with 0.1% FBS at room temperature and treated with drugs for immunostaining.

For primary podocyte outgrowths, glomeruli were seeded onto collagen I‐coated dishes for 4–6 days to allow migration of podocytes. Primary podocytes were then trypsinized and filtered with a 20‐μm cell strainer and re‐plated for experiments.

### 2.1 Ethics Statement

Experiments were performed in accordance with the National Institutes of Health and Institutional Animal Care and Use Guidelines. The Institutional Animal Care and Use Committee at the University of Texas Southwestern Medical Center approved all procedures and protocols. Animals were sacrificed by the i.p. administration of a lethal dose of tribromoethanol (250 mg/kg) and cervical dislocation for tissue collection. We used the ARRIVE reporting guideline to draft this manuscript [12] and the ARRIVE reporting checklist when editing, available from: https://resources.equator‐network.org/guidelines/arrive/arrive‐checklist.docx.

### 2.2 Immunofluorescence Staining of Cells and Glomeruli

Differentiated or primary podocytes were reseeded onto collagen I‐coated glass coverslips and allowed to attach for 24 h, prior to standard fixation and staining procedures. Cells on glass coverslips were then fixed for 10 min at room temperature with 4% paraformaldehyde in PBS, blocked with 3% BSA in PBS for 30 min, and then permeabilized with 0.2% Triton‐100 for 5 min. Staining was accomplished by incubating with primary antibodies overnight at 4°C and then washing three times in PBS before incubation with secondary antibodies for 1 h at room temperature. At the end of the incubations, samples were mounted onto glass slides using mounting medium containing 4′,6‐diamidino‐2‐phenylindole (DAPI). For glomeruli staining, isolated floating glomeruli were maintained in DMEM with 0.1% FBS at room temperature and treated with drugs for 2 h, spun down at 500 g for 5 min, and then washed 3× in PBS. Washed glomeruli were resuspended in PBS and pipetted onto a poly‐prep slide. After glomeruli attached, the slide was rinsed in PBS to remove unattached glomeruli and further fixed with 4% paraformaldehyde for 20 min at room temperature. Fixed glomeruli on slide were then washed 3× in PBS, blocked with SEA BLOCK Blocking Buffer (PIERCE) for 1 h, and then permeabilized with 0.5% Triton X‐100 in PBS for 5 min. Glomeruli on slide were then stained using standard procedures.

### 2.3 Microscopy and Image Analysis

Confocal imaging was performed in the UTSW Nephrology Division Kidney Research Core, on a Zeiss LSM880 with Airyscan laser scanning microscope equipped with Plan‐Apochromat 10×/0.3 NA, 20×/0.8 NA, 25×/0.8 NA, and 63×/1.40 NA oil‐immersion objective (ZEISS, Oberkochen, Germany). Fluorescence images were acquired using ZEN black 2.3 software with a 20×/0.8 NA or 63×/1.40 NA objective, and Zeiss Immersion Oil 518F was used for the 63×/1.40 NA objective. Experiments were performed at constant room temperature. Images or regions of interest (ROIs) were further processed with ZEN 2.6 (blue edition) software.

### 2.4 Scratch‐Induced Cell Migration Assay

Equal numbers of podocytes were seeded on collagen‐coated six well plates. Confluent cell monolayers were washed, and scratch wounds were created with a 1‐mL pipette tip. Culture medium was replaced with culture medium containing DMSO, W11 (1 μM), or FK506 (1 μM). Images were obtained immediately after wound creation and before cell migration. Plates were returned to the incubator, and cells were allowed to migrate for 18‐h, at which time repeat images were obtained. Cell migration rates were calculated from three different regions of the wound width. Average of 10 independent measurements were tracked after 18‐h, and decreased wound width/h was used as an estimate of the cell migration rate.

### 2.5 Immunoblotting

Cultured podocytes after indicated treatments were gently rinsed in PBS and then overlayed with 1 mL 10% TCA/10 mM DTT and snap‐frozen by floating the culture dish on liquid nitrogen. After thawing on bench, cell precipitates were scraped off and collected in a microcentrifuge tube and centrifuged for 1 min at 1000 *g*. Protein precipitates were aspirated free of TCA, washed in 0.5 mL ethyl ether 3 × 15 min each, air‐dried in fume‐hood, and then completely solubilized in 200 uL of 8 M urea, 20 mM Tris (pH 8.6), 23 mM glycine, 10 mM DTT, 4 mM EDTA, and 5% sucrose. Protein concentrations were quantified by Bradford assay (Bio‐Rad). Samples were further reduced in 0.25 LDS sample buffer for SDS‐PAGE per reagent instructions (Thermo Fisher). Separated proteins (2 to 8 μg per lane, depending on protein of interest) were transferred onto a nitrocellulose membrane (Biorad) and then processed for immunoblotting using standard procedures. The amount of protein to load per lane of gel was empirically predetermined by a loading curve comparison for each antibody used.

### 2.6 Co‐Immunoprecipitation Studies for Protein Interaction

Podocytes were cultured on 10 cm collagen‐coated dishes to ~80% confluency and then lysed in RIPA buffer with protease inhibitor (Halt protease inhibitor cocktail, Thermo). Lysate supernatant fractions collected after centrifugation (16 000 rpm, 1 min) were equally divided into two for IP with equal amount of IgG and WNK1 antibodies (control‐IP and WNK1‐IP). To test the effects of WNK1 kinase activity inhibition or activation on protein interactions, cells were pretreated with WNK1‐inhibitor (W11, 1 μM) in culture medium for 3‐h or treated with WNK1‐activator (FK506, 1 μM) in serum‐free medium for 3‐h. Protein G beads (ThermoFisher) were used for immunoprecipitation following the manufacturer's instructions.

### 2.7 Measurement of RLC Phosphorylation

RLC phosphorylation was measured by urea/glycerol‐PAGE as previously described [13]. Podocytes snap‐frozen in TCA and proteins processed in urea sample buffer as detailed above were subjected to urea/glycerol‐PAGE to separate mono‐ and di‐phosphorylated RLC from non‐phosphorylated RLC. Following electrophoresis, proteins were transferred to nitrocellulose membranes and immunoblotted for pan smooth/non‐muscle RLC. The molar ratio of mono‐ or di‐phosphorylated RLC to total RLC was determined by quantitative densitometry of developed immunoblots and expressed as mol phosphate per mol RLC.

### 2.8 Podocyte Cell Culture

Podocyte cell lines were derived from WT and KO primary podocyte outgrowths from glomeruli isolated from adult mice (12–16 weeks of age). Primary podocytes were conditionally immortalized by infection with a lentivirus expressing the temperature sensitive SV40 T antigen tsA58. Lentivirus was produced from pLenti‐SV40‐T‐tsA58 (abm cat# LV629) as previously described [14] using the VSV envelope from pMD2.G (Addgene cat#12259, gift from Didier Trono) and the packaging plasmid pCMV delta R8.2 (Addgene cat # 12263, gift from Didier Trono). Primary glomerular outgrowths were infected for 48 h followed by selection with puromycin (2 ug/ml). Conditionally immortalized mouse podocytes were cultured in RPMI 1640 with 10% FBS. Cells cultures were expanded at 33°C in the presence of interferon, and for experiments, differentiated at 37°C for 7–10 days as previously described [15]. For imaging SLSs, WT podocytes were differentiated in VRAD medium in 37°C for 7 days, following a previously described method [16]. Briefly, the differentiation medium was supplemented with vitamin D3 and retinoic acid to enhance podocyte‐specific gene expression and promote the organization of sarcomere‐like structures, thereby mimicking the phenotype of injured primary podocytes.

## 3 Results

### 3.1 WNK1 Activity Is Required for Maintenance of Podocyte Foot Processes in Glomeruli and in Vivo

**FIGURE 1:**
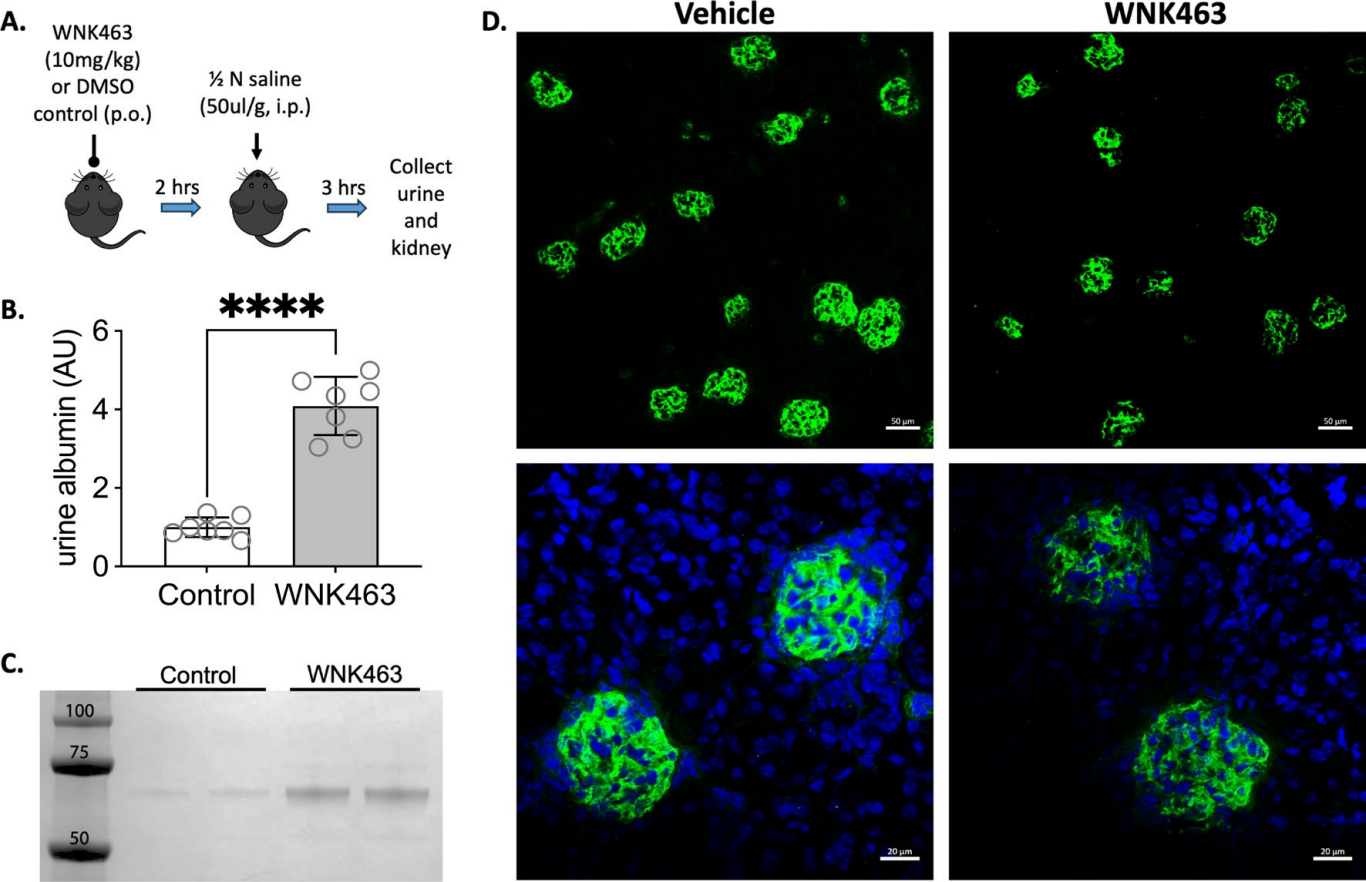
**WNK1 kinase activity is required for maintenance of glomerular function**. (A) Schematic of in vivo acute inhibition of WNK1. WT mice were given WNK inhibitor or equivalent volume of DMSO vehicle by oral gavage (p.o.), 2‐h before i.p. injection of ½ N saline to increase urine output. Urine collected over a 3‐h period were pooled. Kidneys were collected at the end of 3‐h and embedded in OCT. (B) Urine ACR, comparing mice treated with control or WNK463. (C) Coomassie‐stained gel of urinary proteins separated by SDS‐PAGE. Volume loaded was adjusted by creatinine values. (D) Cryosectioned kidneys collected at the end of the study were stained for synaptopodin (green) and nuclei (DAPI, blue). Scale bar: 50 mm (upper row), 20 mm (lower row).

With‐no‐lysine (WNK)1 kinase is expressed in glomerular podocytes and contributes to regulation of glomerular structure [11]. Chemical inhibition of WNK kinases reduced mouse glomerular stiffness, decreased F‐actin, and disrupted podocyte foot process structure, causing a reduction in slit diaphragm density in isolated glomeruli [11]. Conversely, activation of WNK1 kinase by calcineurin inhibitors FK506 or cyclosporin increased glomerular stiffness and F/G actin ratio. As the filtration function of the kidney is dependent on the slit diaphragm structure, we tested the hypothesis that acute inhibition of WNK1 will reduce the filtration function of glomeruli in vivo. WT mice were treated with a WNK kinase inhibitor, WNK463, by oral gavage (10 mg/kg) and then injected with ½ N saline (i.p.) to increase urine flow (Figure 1A). Urine was collected over 3‐h, after which kidneys were collected for immunofluorescence imaging. WNK463 treatment acutely increased mouse urinary albumin levels four‐fold (Figure 1B,C). Cryosectioned kidney cortices stained for synaptopodin showed marked reduction in glomerular staining in mice that were fed WNK463, similar to in vitro WNK463‐treated mouse glomeruli [11]. Podocyte density was not affected by WNK463 treatment (Figure S1A,B). These results show that WNK463 treatment in vivo acutely diminished synaptopodin staining of the glomeruli without affecting the number of podocytes in the glomerulus or synaptopodin protein levels (Figure S1C). Reduction in observed synaptopodin staining may reflect podocyte foot process effacement that coincides with filtration defect.

### 3.2 Differential Expression of NMIIA and NMIIB in Podocyte Foot Processes and Lamellipodial Extensions, Where the WNK1 Kinase Substrates OSR1/SPAK Are Found

Cell structure is determined in part by coordinated activities of actomyosin proteins that drive membrane extensions and cytoskeletal adhesions. Ubiquitously expressed conventional non‐muscle myosin IIs (NMIIs) [17] are the contractile apparatus of the actomyosin cytoskeletal complex where they hydrolyze ATP to convert chemical energy into mechanical functions in cells. NMIIs form bipolar bundles through tail–tail assembly, and bi‐directional myosin ATPase activity drives contractility. Cell polarity is driven in part by the differential distribution of NMII paralogs [18, 19]: NMIIA, NMIIB, and NMIIC. Various reports that support a role for all NMII paralogs in cells, regardless of the amount of protein, raise the importance of understanding the regulatory mechanisms that control NMIIs and underlie the maintenance of podocyte structure [20, 21].

**FIGURE 2:**
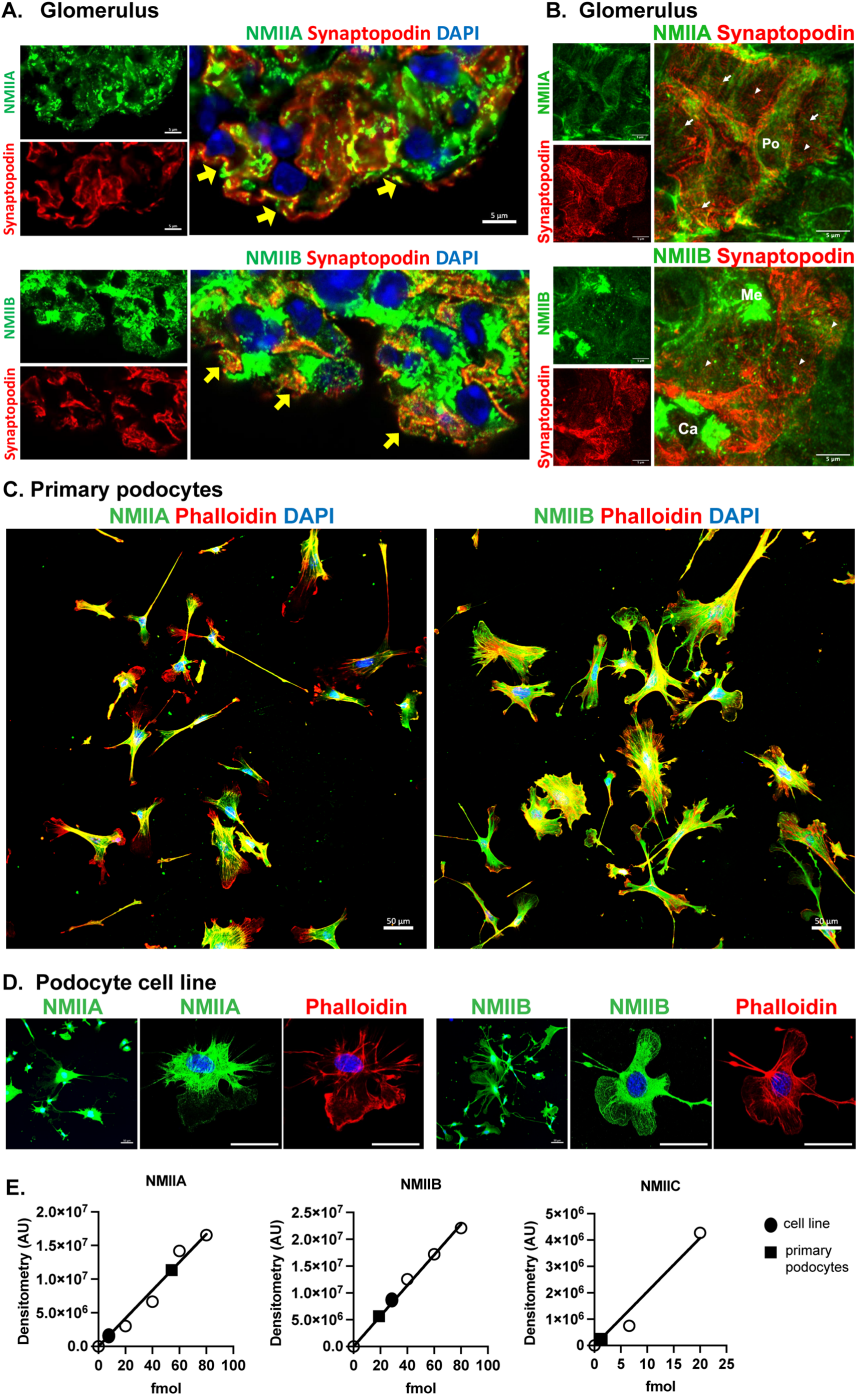
**Distribution of myosin‐IIA/IIB (NMIIA/NMIIB) in mouse glomeruli and isolated podocytes**. (A) Isolated mouse glomeruli were co‐stained for the podocyte marker synaptopodin and NMIIA or NMIIB as indicated to show colocalization in intact mouse glomerular podocytes in situ. The scale bar represents 5 μm. (B) Super‐resolution microscopy of glomerular capillaries in cryosectioned kidney cortex shows colocalization of NMIIA and NMIIB with synaptopodin‐positive major processes (arrows) and foot processes (arrowheads) in podocytes. The scale bar represents 5 μm (C) Isolated primary mouse glomerular podocytes show the differential distribution of NMIIA and NMIIB, where NMIIA is localized centrally in majority of the cells and NMIIB is distributed broadly, including lamellipodial extensions. The scale bar represents 50 μm. (D) Podocyte cell line developed from primary mouse glomerular podocyte outgrowths shows preservation of differential distribution of NMIIA and NMIIB. Scale bar represents 50 μm. (E) Quantification of NMII paralog expression in equivalent amounts of lysates from a WT podocyte cell line (filled circle) and isolated primary podocytes (filled square) by comparison to standard curves generated using purified proteins (open circle). NMIIC was only detectable in primary podocytes.

Based on the dynamicity of podocyte structure in response to WNK1 kinase activity, we hypothesized that NMIIs are downstream effectors of WNK1 kinase activity in podocytes. Using antibodies that were validated with knockout tissues [20, 22], we localized NMIIA and NMIIB in the glomerulus by immunofluorescence microscopy. Although the NMIIA and NMIIB could not be co‐imaged at the same time because the antibodies were from the same species, comparisons of their expression within mouse glomeruli co‐stained for synaptopodin showed NMIIA and NMIIB are both expressed in synaptopodin‐positive regions of the isolated glomerulus (Figure 2A, yellow arrows). Higher magnifications show colocalization of NMIIs in major processes (arrows) and podocyte foot processes (arrowheads) (Figure 2B).

Primary podocyte outgrowths stained for NMIIA and NMIIB showed a distinct pattern of distribution, with NMIIB localized broadly to membrane edges and lamellipodial extensions, while most of NMIIA was centrally located (Figure 2C). These images are consistent with a lesser degree of observed overlap of NMIIA signal with synaptopodin than NMIIB in glomeruli, suggesting that compared to NMIIA, which is known to be more abundant than the other paralogs, a greater proportion of NMIIB is localized to dynamic membrane blebs and extensions. Preservation of the differential distribution of NMIIA and NMIIB in a podocyte cell line developed from WT primary podocyte outgrowths supports the use of podocyte cell lines for studies related to WNK1 and actomyosin signaling (Figure 2D).

To determine whether the total amount of NMII paralogs expressed is a contributing factor for differential localization of NMIIA and NMIIB, we quantified the amount of the protein expressed in podocyte cell lines and primary podocytes by comparison to standard curves using purified proteins (Figure 2E, Figure S2). The calculated molar ratio of NMIIA/NMIIB was 0.17 in the WT podocyte cell line and 3.0 in the primary podocyte. These values show that the primary podocytes have 17× more NMIIA than the developed WT cell line. NMIIC was undetectable in the podocyte cell line by immunoblotting even with 3× greater protein load. The amount of NMIIC/NMIIB in the primary podocytes was 0.03. Thus, the molar ratios of NMIIA:NMIIB:NMIIC is 3: 1: 0.03 in primary podocytes, and 0.17: 1: 0 in the WT podocyte cell line. The differences in amounts of NMIIA:NMIIB and the small amount of NMIIC in the primary podocytes may be from contaminating non‐podocyte cells, which account for < 15% of the total cell number (Figure S8). In both cell preparations, NMIIB comprises a significant proportion of the total NMII paralogs in podocytes, and the expressions of NMIIA and NMIIB are comparable, not orders of magnitude different like it appears to be for NMIIC.

### 3.3 WNK1 Kinase Activity Affects Localization and Activation of NMIIs

**FIGURE 3:**
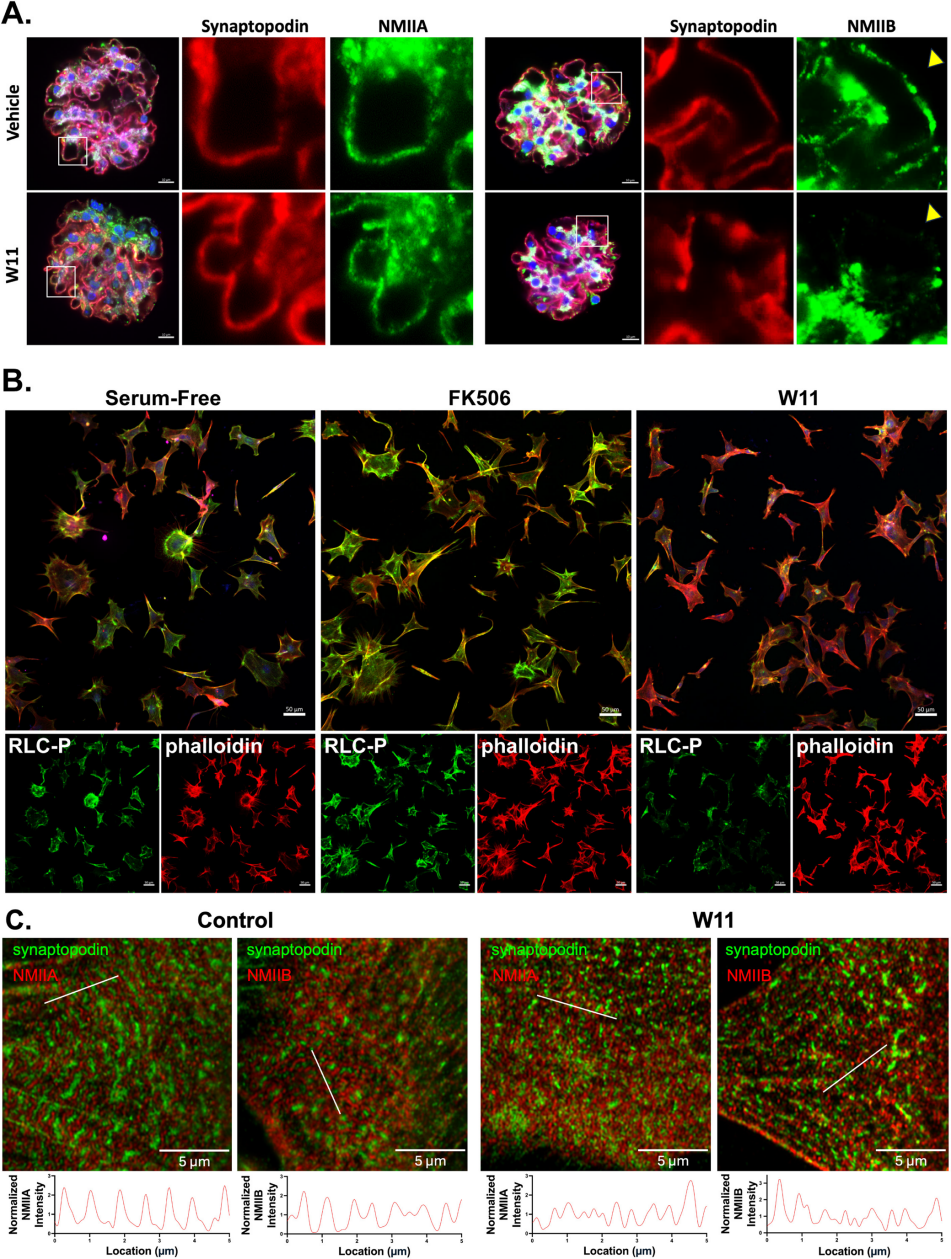
**Acute effects of WNK1 inhibition on NMII paralog localization and actomyosin activation**. (A) Isolated mouse glomeruli after 2‐h treatment with control (vehicle) or 1 μM WNK1‐specific inhibitor (W11), co‐stained for NMIIA/IIB and synaptopodin. First column of images shows merged images of a representative glomerulus, stained as indicated after treatments. Separated channels for the selected region (white box) are shown enlarged. With W11 treatment, NMIIB staining was markedly reduced at many of the glomerular capillary loops (yellow arrowhead), where synaptopodin and phalloidin staining were still present. The scale bar represents 10 μm. (B) Podocytes in culture were stained for phosphorylated RLC (RLC‐P) after 2‐h treatment with FK506 to activate WNK1 kinase or co‐treatment with FK506 and W11 to inhibit WNK1 kinase. Enlarged merged images are shown with separate channels shown below. Scale bar represents 50 μm. (C) Sarcomere‐like structures were induced in WT podocytes with VRAD medium to induce pathologic remodeling processes. The effects of WNK1 inhibition with W11 treatment was assessed by comparison of NMII stain periodicity (lower graphs).

To test the hypothesis that WNK1 kinase activity affects localization of NMII in podocytes, we treated isolated glomeruli with the WNK1‐specific inhibitor WNK‐IN‐11 (W11) [23], and compared the distribution of NMIIs. NMIIB co‐localization with synaptopodin in the capillary loops was markedly reduced in many areas, while NMIIA co‐localization was not as affected (Figure 3A). These results suggest that while NMIIA and NMIIB are both present in podocytes, NMIIB localization at synaptopodin‐positive areas may be affected to a greater extent than NMIIA by WNK1 inhibition. Pearson's correlation coefficients were 0.26 for NMIIA and 0.38 for NMIIB (Figure S3A).

As NMIIs are ATPases that are activated by phosphorylation of their regulatory light chain (RLC), we evaluated whether WNK1 inhibition not only affected the localization of NMIIs but also their activity. WT‐cultured podocytes were treated with the calcineurin inhibitor FK506, which we showed increased WNK1 kinase activity, or FK506 and WNK1‐inhibitor W11 to test whether FK506‐induced changes can be attributed to WNK1 activation. Staining of the cells for phosphorylated RLC (RLC‐P) after treatments showed FK506 significantly increased RLC‐P over serum‐free conditions (Figures S3B, S4A). Co‐treatment with W11 significantly reduced RLC‐P levels below that of serum‐free conditions (Figure 3B, Figure S3B), indicating that in cultured podocytes, NMIIs have a high basal activity level that is reduced by WNK1 inhibition. These results suggest WNK1 activity contributes to the maintenance of baseline NMII activity.

NMIIA forms “sarcomere like structures” (SLSs) with an alternating synaptopodin staining pattern in the podocytes of glomerular injury models and in cultured podocytes after VRAD medium‐induced differentiation, which simulate injured podocytes [24, 25]. We asked whether WNK1 activity contributed to the maintenance of SLSs by comparing the distribution of NMIIA and NMIIB in response to W11 treatment. We found that synaptopodin and NMIIA (Figure S5) and NMIIB (Figure S6) appeared more diffuse in response to W11, with disruption of SLS periodicity, indicating disruption of the NMII structures with WNK1 inhibition (Figure 3C). Thus, the activity of WNK1 contributes to baseline NMII activity in podocytes, at the level of cell membrane extensions and SLS formation in injury.

### 3.4 WNK1 Is Implicated in the Pathogenesis of Alport Syndrome

**FIGURE 4:**
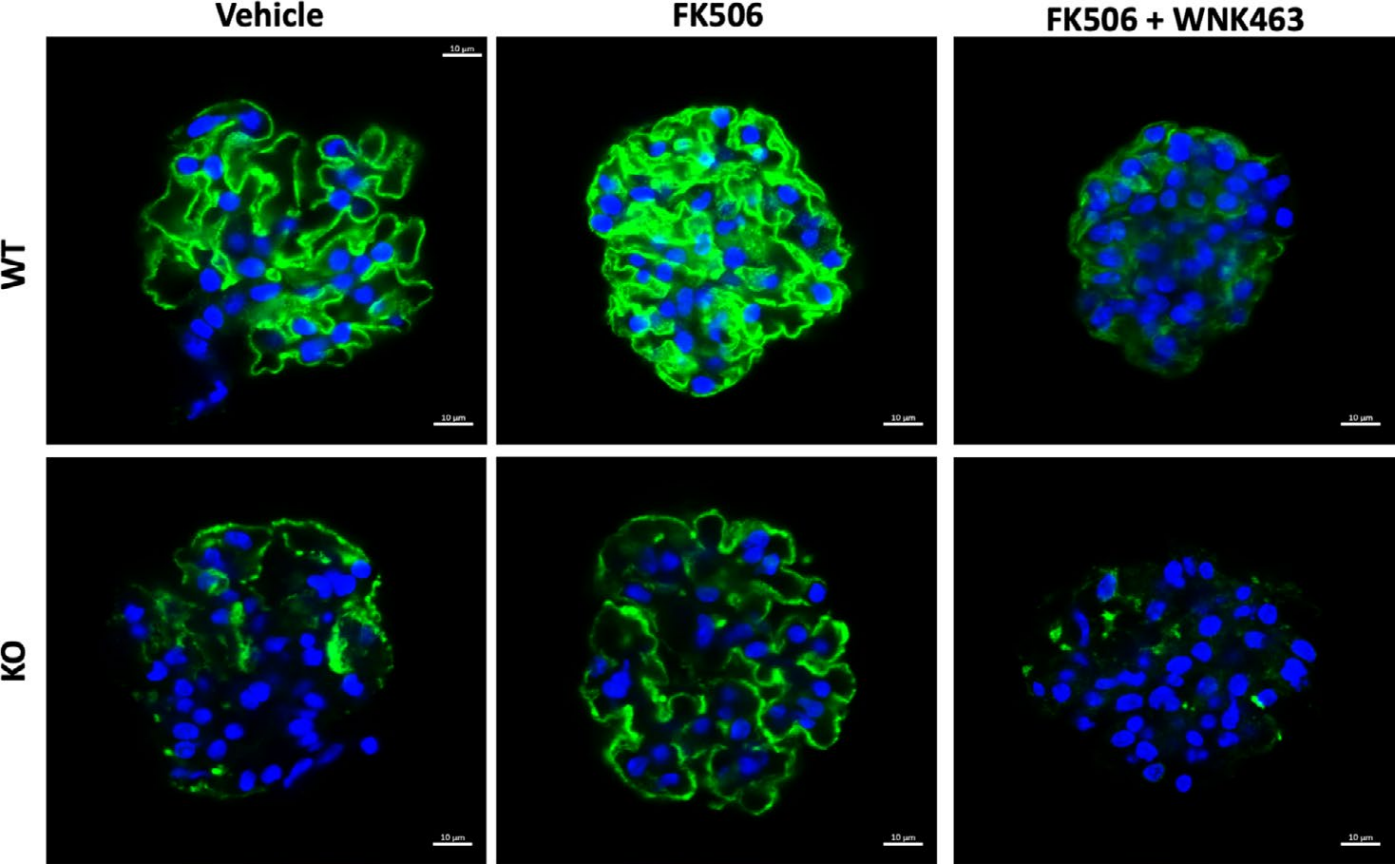
**WNK1 kinase signaling directly regulates podocyte foot process structure in situ. (**A) Representative synaptopodin (green) and DAPI (blue) stained glomeruli from WT (upper row) and ***Col4a3*** ^***−/−***^ Alport knockout (KO, lower row) mice after ex vivo treatments as indicated.

Podocytes in models of glomerular disease such as Alport Syndrome have reduced cell projections, resulting in foot process effacement [11, 26]. Although the specific mechanisms are unclear, based on prior evidence using WT podocytes, we hypothesized that FK506 could contribute to retention of podocytes during disease progression by restoring podocyte structure, including foot processes through activation of WNK1 kinase. We confirmed drug efficacy using cultured WT podocytes (Figure S4) and then treated isolated glomeruli from the WT or *Col4a3* ^*−/−*^ model of Alport Syndrome (KO). Staining of podocyte‐specific synaptopodin after in vitro treatment with vehicle or FK506 in combination with WNK463 showed that WNK1 activity was required for continuity of glomerular capillary synaptopodin staining (Figure 4, upper row). KO glomeruli, which have decreased podocyte number [26], had discontinuous and reduced synaptopodin staining that could be improved with acute FK506 treatment. WNK463 blocked the improvement associated with FK506 treatment and further diminished the intensity of synaptopodin staining, suggesting WNK kinase inhibition further disrupted the glomerular structure (Figure 4, lower row). These results support the hypothesis that WNK1 kinase activity is a necessary and tractable kinase for maintenance of podocyte structure.

### 3.5 Podocyte Injury Phenotype in Alport Syndrome Includes WNK1 and Actomyosin Protein Expression Differences

In the *Col4a3* ^*−/−*^ (KO) Alport Syndrome model (C57BL6/J background), the disease progresses relatively slowly with mice succumbing from renal disease at around 8 months of age [27]. Thus, earlier time points, 2–4 months, represent early stages of renal disease, where injured podocytes are viable and renal function is preserved. Comparison of glomerular cell outgrowths from 4‐month‐old WT mice showed uniform podocyte cell shape and size (Figure S7A), but heterogeneity in size and cell number in the KO glomerular cell outgrowths (Figure S7B). Cells that migrated from decapsulated glomeruli were positive for the podocyte‐specific marker WT1 (Figure S8), confirming that they are podocytes (> 85% of total). The observed differences in cell migration and heterogeneity in cell shape are consistent with the known pathology of glomerular diseases, where injured podocytes are broadly distributed throughout the glomeruli in the renal cortex.

**FIGURE 5:**
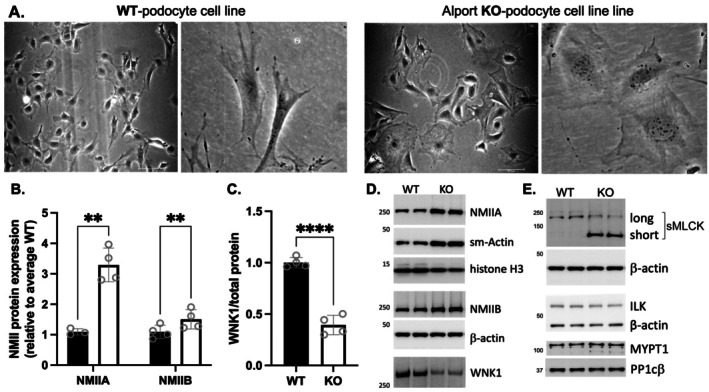
**Characterization of podocyte cell lines developed from WT and *Col4a3*** ^***−/−***^ **Alport mouse glomeruli**. (A) Live cell microscopy of podocytes in culture. (B) Comparison of NMIIA and NMIIB expression showed both were significantly increased in KO (white bar) over WT (black bar). (C) WNK1 expression was significantly reduced in KO samples. Each dot represents average of triplicate experiments performed 3–4 distinct times and immunoblotted separately. Bars represent data normalized to mean of WT ± S.D. **p < 0.01 by 2‐way ANOVA, followed by Tukey's multiple comparisons post‐test, ****p < 0.0001 by t‐test, 2‐tailed, using GraphPad Prism. (D) Representative immunoblots of actomyosin proteins and WNK1. (E) Representative immunoblots of selected regulators of NMII activity.

Extending our studies to biochemical characterization of WNK1‐mediated signaling to NMIIs, we generated cell lines from WT and KO primary podocytes (Figure 5A) that retained distinct morphological features. Live cell microscopy shows KO podocytes had fewer lamellipodial extensions, and many appeared polygonal in shape with reduced structural complexity. The expression of WNK1, actomyosin proteins, and canonical regulators of NMII contractile activity were compared. NMIIA and NMIIB protein expression showed significant upregulation in KO cells, respectively by 3.3 ± 0.6 fold and 1.5 ± 0.3 fold (Figure 5B,D). Smooth muscle actin was also significantly increased in KO cells (2.4 ± 0.5 fold, *N* = 4) compared to WT (Figure 5D), indicating activation of contractile protein expression. WNK1 protein was significantly reduced in the KO cells (0.4 ± 0.1 fold, *N* = 4) compared to WT in both cell lines (Figure 5C,D) and primary cells from 2.5‐month‐old mice (Figure S7). Despite the lack of significant lamellipodial features, more NMIIB along membrane edges was observed in KO podocytes (Figure S9B), where the specific substrate of WNK1 kinase, OSR1, was shown to be phosphorylated in response to FK506 [11] (Figure S4). These results support the hypothesis that WNK1 activity at podocyte membrane edges may contribute to both NMIIA‐ and NMIIB‐mediated membrane dynamics.

The canonical regulators of NMII ATPase reactions that drive contractions in non‐muscle cells are the Ca^2+^/CaM‐dependent myosin light‐chain kinase (MLCK) and the myosin light‐chain phosphatase (MLCP). The gene for MLCK in non‐muscle cells, *MYLK*, encodes 3 distinct proteins: long non‐muscle MLCK, short smooth muscle MLCK, and telokin [28, 29]. Non‐muscle MLCK is dominantly expressed in the kidney, consistent with observed enrichment in other non‐muscle cells [30]. The MLCP that regulates inactivation of NMIIs is a holoenzyme comprising the regulatory myosin target subunit MYPT1, a catalytic subunit PP1c, and an accessory protein M21 [31]. MYPT1 is ubiquitously expressed and targets the catalytic subunit PP1c to myosin through high affinity binding sequences to dephosphorylate RLC. Expression levels of these regulatory proteins, basal activation states, and localization within normal and diseased glomerular podocytes have not been clearly documented.

The expressions of selected regulators of NMII contractile activity, smMLCK, and the subunits that form the MLCP were compared between WT and KO podocytes (Figure 5E). WT podocytes expressed only non‐muscle MLCK (Figure 5E), but KO podocytes showed increased expression of the smooth muscle‐specific short smMLCK isoform. The total smMLCK expressed (long + short form) was significantly higher in KO cells, which expressed a 3.7 ± 1.8 fold greater amount of total MLCK (Figure 5E). The expression level of interleukin‐linked kinase (ILK), which is found in nascent and mature focal adhesions and can also phosphorylate RLC, was not different between WT and KO. Surprisingly, the protein levels of MYPT1 and the catalytic subunit PP1cβ were also comparable between WT and KO cells, despite increases in the substrate and kinase amounts. Thus, compared to WT, KO podocytes have increased smMLCK, smooth muscle actin, as well as total NMIIA and B. These measurements suggest changes in actomyosin protein expression occur very early in the disease process, and that greater substrate availability for smMLCK in the KO podocytes may be a compensatory mechanism to retain podocyte traction force.

### 3.6 Activation of NMII Is Affected by WNK1 Activity in WT and KO Podocytes

**FIGURE 6:**
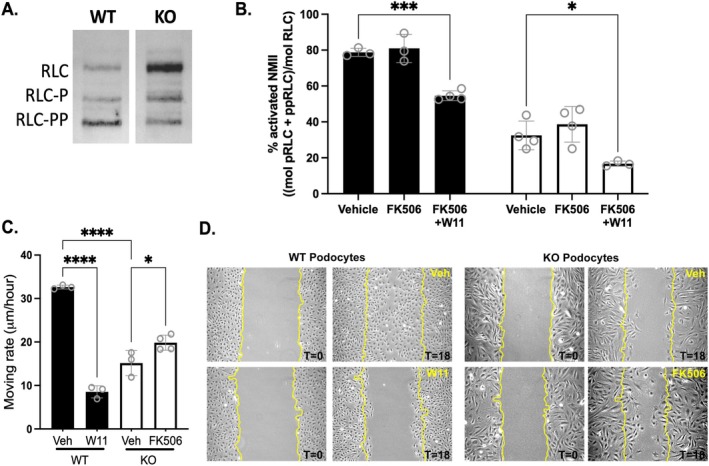
**Effects of WNK1 activity on levels of activated NMII and cell migration**. (A) Representative immunoblot of WT and KO podocyte cell extract for myosin RLC after separation of mono‐ (RLC‐P) and di‐phosphorylated (RLC‐PP) forms from non‐phosphorylated RLC. Relative band intensities show that a greater amount of total RLC is di‐phosphorylated (bottom band, RLC‐PP) in WT podocytes, whereas a greater amount is unphosphorylated and inactive (top band, RLC) in KO. (B) Graphed measurement of total activated NMII in WT and KO podocytes after indicated treatments, determined by calculated % of total RLC that is mono‐ or di‐phosphorylated. Black bars are WT, white bars are KO. *p < 0.05, ***p < 0.001 by 2‐way ANOVA, followed by Dunnett's multiple comparisons post‐test using GraphPad Prism. (C) Effects of WNK1 activity on migration properties of WT and *Col4a3* ^*−/−*^ Alport KO podocytes. Comparisons of quantified cell migration rates, as a measure of wound healing capability, are shown. Dots represent average wound width measured in triplicate, from separate experiments; *N* = 3 or 4. Significance was determined by ordinary one‐way ANOVA, followed by Bonferroni's multiple comparisons test using GraphPad Prizm; *p < 0.05, ****p < 0.0001. (D) Representative images using the scratch wound‐healing assay to induce podocyte migration. Cells were grown to confluency determined by dish surface area coverage. KO podocytes are larger and covered a greater surface area than WT podocytes (Figure 3A). Images were taken immediately after scratch wound (T0) and 18 h after culture in normal FBS‐supplemented media (T18), in the presence of WNK1 inhibitor or activator as indicated. WT podocytes subjected to WNK1‐inhibition (W11) had a significantly reduced capacity to migrate into the wound area compared to vehicle control (Veh). Compared to WT at 18 h, Alport KO‐podocytes had a significantly reduced migration capacity under normal culture conditions (Veh). WNK1 activation with FK506 modestly increased the wound healing rate. The scale bar represents: 100 μm.

Reduced colocalization of NMIIB and synaptopodin in podocytes on glomerular capillaries in response to WNK1 inhibition (Figure 3A) suggests that NMIIB activation is sensitive to WNK1 activity. NMII activation is dependent on the phosphorylation of RLC at Ser‐19 (RLC‐P) or Thr‐18/Ser‐19 (RLC‐PP) [32]. Based on increased NMIIs and smMLCK, we asked whether KO podocytes have more NMII activated by phosphorylation. Baseline comparison of activated NMII in WT and KO podocytes in culture showed that most of the NMII proteins in WT are activated by phosphorylation, while most of the NMII proteins in KO were inhibited (Figure 6A,B).

While comparison of mono‐phosphorylated RLC (RLC‐P) was significantly increased by FK506 treatment (Figure 3B, S3B, S4B), activation of WNK1 by FK506 did not induce a significant increase in the total activated pool of NMII, as determined by the sum of RLC‐P and RLC‐PP. Addition of W11 in addition to FK506 reduced the extent of activated NMII by about 20% in both WT and KO, where the total activated NMII was reduced from 81% ± 8% to 55% ± 3% in WT and from 39 ± 10 to 17% ± 1% in KO. These results suggest that WNK1 activity contributes to regulation of about 20% of total NMIIs in both WT and KO podocytes, presumably at the membrane edges of lamellipodial extensions where WNK1 appears to be most active [11].

Based on our immunoblot comparisons, KO podocytes have nearly 3‐fold greater total NMII, and most of the myosins are in the inactive state, despite greater expression of total smMLCK (Figure 5A,B). We asked whether WNK1‐activation by FK506 was sufficient to augment podocyte lamellipodia formation in injured podocytes despite lower levels of activated NMII. Scratch wound healing assays using WT and KO podocytes showed that in the presence of FBS (Veh), WT podocytes could migrate to fully cover bare zones of the dish within 18 h (Figure 6D). Inhibition of WNK1 with W11 over the same time frame significantly reduced the migration rate of WT podocytes by over 3‐fold from 33 ± 0.5 mm/h to 8.5 ± 1.4 mm/h, indicating that WNK1 activity is necessary for normal podocyte migration (Figure 6C,D), consistent with observations previously made using a pan‐WNK inhibitor and WNK1 siRNA [11]. Compared to WT, KO podocytes had a significantly reduced rate of migration under FBS‐supplemented conditions (Figure 6C,D), consistent with low extent of activated NMII (Figure 6A,B). Activation of WNK1 in KO podocytes using FK506 augmented the migration rate from 15 ± 0.7 mm/h to 20 ± 0.9 mm/h (Figure 6C,D). Although the increase is modest, the results indicate that diseased podocytes, like those from Alport KO mice, can be treated to activate NMIIs at leading edges, suggesting it could improve slit diaphragm structure, consistent with observed improvement in synaptopodin immunofluorescence in isolated glomeruli that had been treated with FK506 (Figure 4). These results collectively show that NMII activity is partly regulated by WNK1 activity. Thus, although the amount of NMIIA and NMIIB at the membrane cell extensions is a small proportion of the total and the amount of WNK1 kinase was further reduced in the KO cells, increased WNK1 activity was sufficient to augment podocyte cell motility in injured podocytes.

### 3.7 WNK1 Kinase Augments Podocyte Membrane Extensions Through Activity‐Dependent Signaling Complexes

**FIGURE 7:**
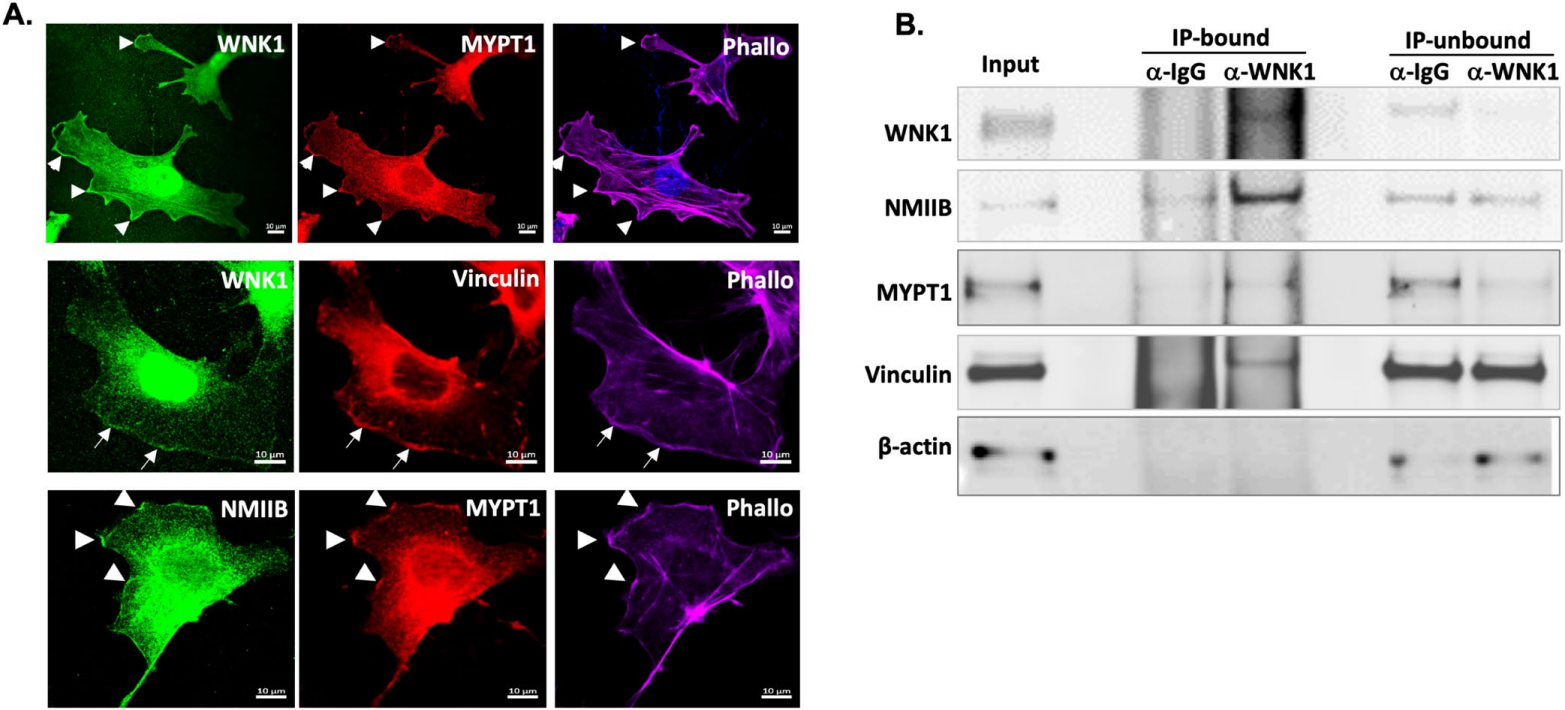
**Evidence of WNK1 kinase interaction with actomyosin and focal adhesion proteins. (**A) Representative images of WT podocytes showing colocalization of WNK1 with MYPT1 (upper row), WNK1 with vinculin (middle row) and NMIIB with vinculin (bottom row). Arrowheads and arrows point to membrane undulations where WNK1 kinase appears to be enriched. The scale bar represents: 10 μm. (B) Immunoblot of selected proteins that co‐immunoprecipitated with WNK1 antibody. Compared to IgG control, NMIIB, MYPT1, and vinculin all co‐IP selectively with WNK1 kinase. Reduction in the unbound fraction was seen for WNK1 and MYPT1, but not for NMIIB and vinculin, consistent with colocalization studies in panel A that shows a small proportion of the total is found colocalized with WNK1 at the membrane edges. β‐Actin was used as a control for loading and indicator of IP‐sample washing efficiency. Selected immunoblots from multiple co‐IP experiments are shown.

During glomerular disease progression, effacement of podocyte foot processes [26] precedes detachment from the glomerular basement membrane, despite compensatory increases in SLSs that contribute to adhesion [25]. As shown above, although WNK1 protein is reduced in Alport KO podocytes (Figure 5C,D), cell migration is augmented by WNK1 activation‐mediated NMII activity (Figure 6C,D). Given the comparatively greater increase in NMIIA than NMIIB in the KO podocytes (Figure 5B), we asked whether the modest increase in NMIIB expression contributes to WNK1 kinase activity‐mediated effects on podocyte motility. Using IF co‐staining experiments, we found that WNK1, MYPT1, NMIIB, and vinculin are enriched in and colocalize at undulating active membrane edges (Figure 7A). To test the hypothesis that these proteins physically interact, we performed co‐IP experiments with anti‐IgG control or anti‐WNK1 antibodies and immunoblotted for the proteins indicated (Figure 7B). The results show that WNK1, NMIIB, MYPT1, and vinculin all selectively co‐IP with WNK1 over control IgG.

**FIGURE 8:**
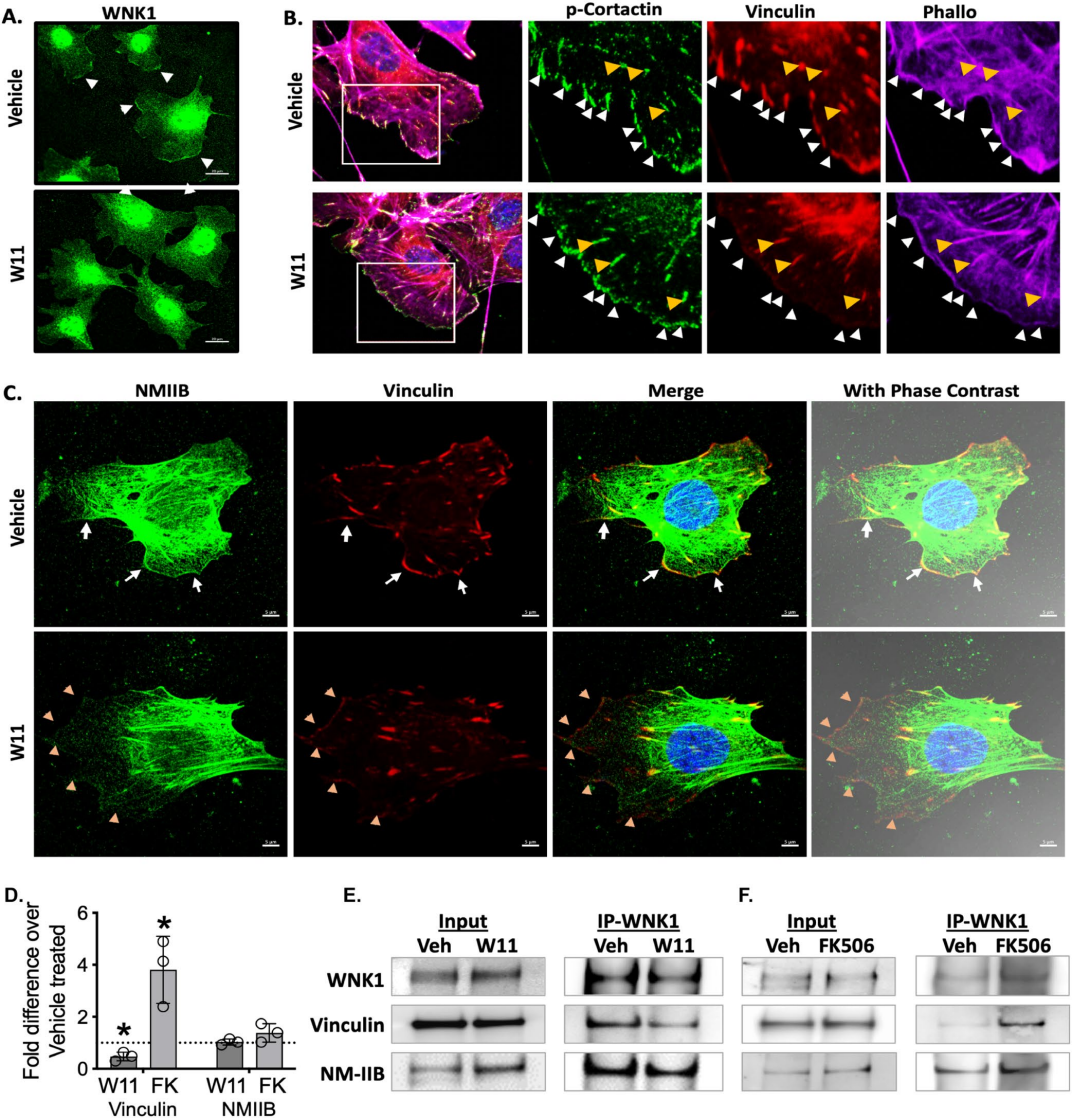
**Membrane distribution of vinculin is decreased with WNK1 activity inhibition**. (A) WNK1 staining in podocytes shows WNK1 kinase localization to membrane edge undulations (arrowheads) was decreased from spread membrane with WNK1 kinase activity inhibition by W11 treatment. The scale bar represents: 20 μm. (B) Effects of WNK1 kinase inhibition on co‐localization of vinculin with p‐cortactin (used as lamellipodia marker). Decreased vinculin from edge of lamellipodia (white arrows) but not cytoplasmic vinculin (yellow arrows) are indicated in enlarged images. (C) Vinculin and NMIIB both decreased from the leading edge of lamellipodia with WNK1 activity inhibition, but not in the cytoplasm, where strong colocalization is evident in mature focal adhesions. White arrowheads in control points at leading edges of cells where undulating membrane edges show NMIIB colocalized with vinculin. Yellow arrowheads in W11‐treated cells point at the membrane edges with evident decreases in both NMIIB and vinculin. The scale bar represents: 5 μm. (D) Comparison of vinculin and NMIIB protein co‐immunoprecipitated with WNK1. *p < 0.05 by T‐test using GraphPad Prism. (E) Representative immunoblots of NMIIB, vinculin, and WNK1 in WNK1‐IP samples from podocytes treated with vehicle or WNK1‐inhibitor (W11 1 μM). (F) Representative immunoblots of NMIIB, vinculin, and WNK1 in WNK1‐IP samples from the podocytes treated with vehicle or WNK1‐activitor (FK506 1 μM).

Based on evidence of WNK1 kinase activity regulation by FK506 that increases cell motility (Figure 6C,D) and interaction with key focal adhesion proteins, NMIIB and vinculin (Figure 7B), we hypothesized that WNK1 activity is a determinant of podocyte membrane extension through signaling to NMIIB and vinculin at the cell membrane and by extension, foot processes. Analysis of the podocyte cell membrane at leading lamellipodial extensions shows WNK1 kinase activity is required for the observed undulating pattern of WNK1 at the membrane edges (Figure 8A). Inhibition of WNK1 kinase with W11 caused a decrease in vinculin staining at lamellipodial edges where phosphorylated cortactin is found (Figure 8B). The WNK1 activity‐dependent enrichment of vinculin at membrane edges was coincident with NMIIB localization (Figure 8C), which was also reduced with WNK1 kinase inhibition. These observations are consistent with colocalization and co‐IP studies using podocytes in culture (Figure 7) that showed evidence of WNK1 kinase interaction with NMIIB and vinculin.

To demonstrate that WNK1 kinase activity contributes to the association of focal adhesion complex proteins, co‐IP was performed using stimulated podocytes (10% FBS) under WNK1‐inhibited (W11) or WNK1‐activated conditions (FK506). Comparison of immunoprecipitated proteins showed that a greater proportion of vinculin co‐IPed with activated WNK1 kinase than with inhibited WNK1 kinase (Figure 8D–F). NMIIB (Figure S8A) and vinculin (Figure S8B) staining are both reduced with WNK463 treatment, consistent with results from podocytes in culture (Figure 8B,C). Collectively, these studies support the hypothesis that WNK1 kinase activity contributes to the regulation of podocyte membrane dynamics through association with focal adhesion proteins, which include not only NMIIA but also activated NMIIB.

## 4 Discussion

In several studies, CNIs, cyclosporin A and tacrolimus (FK506) reduced proteinuria in patients with nephrotic syndrome [33, 34, 35, 36, 37, 38, 39]. Mouse models of nephrotic syndrome suggest that CNIs exert their podocyte protective effects through suppression of inflammatory responses as well as non‐immune‐mediated mechanisms of actin including synaptopodin stabilization [40, 41]. However, the reported effects of CNIs on podocytes were empirical in nature without definition of causal mechanisms. Our studies present WNK1 kinase activity‐dependent maintenance of podocyte foot processes as a potential mechanism involving vinculin and actomyosin activity that can be acutely and directly manipulated by CNIs.

Using podocyte cell lines, primary podocytes, as well as isolated glomeruli, we found the differential distribution of NMIIA and NMIIB in healthy and diseased podocytes from an Alport model. Our novel discovery of a distinct pool of NMIIB present at the edge of lamellipodial extensions and evidence of a WNK1‐NMIIB signaling axis in glomerular podocytes support the hypothesis that WNK1 activity contributes to the regulation of cell motility and membrane extensions in part by signaling not only to NMIIA but also NMIIB.

Early manuscripts describing podocytes in culture showed different podocyte cell shapes that were attributed to culture conditions [42, 43]. We found that WT and Alport KO primary podocytes under matched conditions have distinct cell shapes that are similar to those found in our conditionally immortalized WT and KO cell lines that were expanded in the presence of interferon at 33°C, and differentiated at 37°C. These differences in podocyte structure and behavior represent the effects of the different biologic processes associated with normal versus Alport syndrome environments. Persistence of differences in culture after the cells leave the glomerular capillaries indicates that the KO podocytes have intrinsic differences from WT cells that are characteristic of their disease state and independent of external factors such as interactions with other cells, the glomerular basement membrane, or culture conditions.

In non‐muscle cells, NMII activity is regulated by phosphorylation of its RLC subunit. Re‐distribution of the NMII fluorescence signal in response to WNK1 inhibition in intact glomeruli suggests that the myosin network is in part regulated by WNK1 kinase‐mediated activation of a kinase for RLC or inhibition of MLCP. MLCP activity inferred from measurements of phosphorylated MYPT1/total MYPT1 in podocytes treated with W11 were not significantly different, suggesting reduction in activated myosin in response to W11 is attributed to reduced phosphorylation of RLC. Several kinases have been shown to phosphorylate RLC in non‐muscle cells, including ILK, DAPKs, ROCK, as well as the canonical MLCK. Notably, two splice variants of MLCK are expressed in primary podocytes and podocyte cell lines, the long non‐muscle MLCK, and the shorter smooth‐muscle MLCK [28]. While in cultured cell lines, long MLCK is often exclusively expressed [29], we discovered that in KO podocytes, the short smooth muscle MLCK is the dominant variant. This was an unexpected finding, given that most contractile smooth muscle cells rapidly de‐differentiate in culture, cease to express the shorter smooth muscle MLCK, and re‐express the long variant. Long and short MLCK have comparable catalytic rates [29], and the region of the kinase that is expressed in the long form partly contributes to localization of the kinase during mitosis [44]. RNAseq analysis of differentially expressed genes in primary podocytes from WT and Alport KO mice showed over‐representation of genes associated with the Lysosome, Focal Adhesion, and Cell‐Substrate Junction (Figure S12) and increased expression of MRTFa/b regulated genes that are associated with myofibroblast differentiation [45], TAGLN (2.5‐fold increase in KO), ACTA2 (9.5‐fold increase in KO), consistent with the KO podocyte cell line that showed increased smooth muscle actin expression (Figure 5D). Whether re‐expression of the short smooth muscle MLCK in disease‐model podocytes reflects compensatory responses to alter shape or to retain strong cell–cell or matrix attachments, and whether this change represents a broader change in gene expression profile remains to be investigated.

NMIIs have two phosphorylation sites on the RLC that regulate myosin ATPase activity. The physiological significance of di‐phosphorylated RLC is unclear, given that it does not augment mono‐phosphorylated myosin ATPase activity and is not found at significant levels in activated smooth muscle tissue. Several mechanisms of NMII regulation in podocytes have been described by others [46, 47, 48] that act through known regulators of NMIIs, MLCK and ROCK. MLCK and ROCK differentially activate peripheral and central pools of NMIIs in cultured cells to respectively promote mono‐ and di‐phosphorylation of RLC [49]. This pattern is reminiscent of the central distribution of NMIIA and peripheral or extension‐associated distribution of NMIIB in primary and cultured WT and KO podocytes (Figure 2, S8). A significant increase in mono‐phosphorylated RLC (RLC‐P) in response to FK506 treatment suggests WNK1‐mediated activation of MLCK. Recently, WNK1 activity was shown to be regulated by Piezo1 [50], a mechanosensitive ion channel that affects levels of intracellular Ca^2+^. Given that MLCK is Ca^2+^/CaM‐dependent, our results are consistent with Ca^2+^ dynamics being a component of WNK1 signaling.

Pathologic remodeling of podocytes includes increased expression of NMIIA (Figure 5A) and the appearance of SLSs patterning with synaptopodin in situ [25], which can be replicated in both primary podocytes and in podocyte cell line after differentiation in VRAD‐media [24]. Interestingly, we found that NMIIB also forms SLSs like NMIIA. A modest increase in NMIIB that we observed (Figure 5A) may have been undetected in prior immunofluorescence imaging by others due to differences in fixation methods [51] and obscuring of low‐intensity signals in the podocytes by higher‐intensity signals from endothelial cells that express greater amounts of NMIIB, necessitating focused imaging of capillaries (Figure 3A, S11). The collective evidence in the current work augments the importance of both NMIIA and NMIIB, which have received relatively little attention in studies of podocyte cell biology.

NMIIA and NMIIB are both expressed in glomerular podocytes, and based on comparative studies of podocyte cell morphology and function, they are important determinants of glomerular stiffness, a characteristic that reflects glomerular capillary and podocyte integrity [1, 11]. Moreover, WNK1 contributes to the regulation of vinculin and NMIIs to affect podocyte cell migration and contractility, reflective of podocyte membrane and actomyosin dynamics in situ, on glomerular capillaries. The disruption of SLS patterning after inhibiting WNK1 further supports a link between the WNK1 pathway and NMIIs in the pathological remodeling of injured podocytes. The cytoskeletal proteins, myosins, and the regulated signaling by WNK1, MLCK, and MLCP involved in membrane dynamics in vitro may reflect the NMIIs, actin, and foot process structures in vivo. Thus, our findings suggest that reported improved GFR in response to CNIs may be partly attributed to NMII activity and membrane WNK1‐NMIIB signaling axis in podocytes [26], and NMIIB activation may be targetable in early stages of podocyte injury to delay or reverse foot process effacement and podocyte loss in glomerular diseases.

Limitations of our studies include the acute nature of observed effects, which correlate well with in vitro studies presented, but may not be reflective of the pathophysiology in chronic conditions like Alport syndrome and other CKD. While beyond the scope of this manuscript, further studies using genetic approaches are needed to address the physiological significance of WNK1 signaling at the slit diaphragm.

## Author Contributions

Z.L. and A.N.C. conceived and designed the research; Z.L., E.L., S.J., F.A., J.Y., and A.N.C. performed the research and acquired the data; Z.L., S.J., and A.N.C. analyzed and interpreted the data; M.A.R., P.R., L.A.B., and R.T.M. provided critical reagents; Z.L. and A.N.C. drafted the manuscript; Z.L., L.A.B., H.Y.S., R.T.M., and A.N.C. contributed to revising the manuscript.

## Funding

This work was supported by HHS | NIH | National Heart, Lung, and Blood Institute (NHLBI), HL146757.

HHS | NIH | National Institute of Diabetes and Digestive and Kidney Diseases (NIDDK), DK139111, DK083592, DK131177, DK141178.

## Conflicts of Interest

The authors declare no conflicts of interest.

## Supporting information


**Data S1:** Supplementary Figures.

## Data Availability

Included in article.

## References

[1] A. E. Embry , Z. Liu , J. M. Henderson , et al., “Similar Biophysical Abnormalities in Glomeruli and Podocytes From Two Distinct Models,” J Am Soc Nephrol 29 (2018): 1501–1512. 29572404 10.1681/ASN.2017050475 PMC5967771

[2] R. Verma , M. Venkatareddy , A. Kalinowski , et al., “Nephrin Is Necessary for Podocyte Recovery Following Injury in an Adult Mature Glomerulus,” PLoS One 13 (2018): e0198013. 29924795 10.1371/journal.pone.0198013 PMC6010211

[3] M. Nagata , “Podocyte Injury and Its Consequences,” Kidney International 89 (2016): 1221–1230. 27165817 10.1016/j.kint.2016.01.012

[4] F. Ding , L. Wickman , S. Q. Wang , et al., “Accelerated Podocyte Detachment and Progressive Podocyte Loss From Glomeruli With Age in Alport Syndrome,” Kidney International 92 (2017): 1515–1525. 28754557 10.1016/j.kint.2017.05.017 PMC5696060

[5] J. E. Wiggins , M. Goyal , S. K. Sanden , et al., “Podocyte Hypertrophy, “Adaptation,” and “Decompensation” Associated With Glomerular Enlargement and Glomerulosclerosis in the Aging Rat: Prevention by Calorie Restriction,” J Am Soc Nephrol 16 (2005): 2953–2966. 16120818 10.1681/ASN.2005050488

[6] C. N. Frank , X. Hou , A. Petrosyan , et al., “Effect of Disease Progression on the Podocyte Cell Cycle in Alport Syndrome,” Kidney International 101 (2022): 106–118. 34562503 10.1016/j.kint.2021.08.026

[7] A. D. Doyle , S. S. Nazari , and K. M. Yamada , “Cell‐extracellular matrix dynamics,” Physical Biology 19 (2022): 021002. 10.1088/1478-3975/ac4390 PMC8855216 34911051

[8] A. D. Doyle , N. Carvajal , A. Jin , K. Matsumoto , and K. M. Yamada , “Local 3D Matrix Microenvironment Regulates Cell Migration Through Spatiotemporal Dynamics of Contractility‐Dependent Adhesions,” Nature Communications 6 (2015): 8720. 10.1038/ncomms9720 PMC4643399 26548801

[9] P. Kanchanawong and D. A. Calderwood , “Organization, Dynamics and Mechanoregulation of Integrin‐Mediated Cell‐ECM Adhesions,” Nature Reviews. Molecular Cell Biology 24 (2023): 142–161. 36168065 10.1038/s41580-022-00531-5 PMC9892292

[10] A. Greka and P. Mundel , “Cell Biology and Pathology of Podocytes,” Annual Review of Physiology 74 (2012): 299–323. 10.1146/annurev-physiol-020911-153238 PMC3600372 22054238

[11] Z. Liu , “Control of Podocyte and Glomerular Capillary Wall Structure and Elasticity by WNK1 Kinase,” Frontiers in Cell and Developmental Biology 8 (2020): 618898. 33604334 10.3389/fcell.2020.618898 PMC7884762

[12] N. Percie du Sert , V. Hurst , A. Ahluwalia , et al., “The ARRIVE Guidelines 2.0: Updated Guidelines for Reporting Animal Research,” PLoS Biology 18 (2020): e3000410. 32663219 10.1371/journal.pbio.3000410 PMC7360023

[13] E. Lee , Z. Liu , N. Nguyen , A. C. Nairn , and A. N. Chang , “Myosin Light Chain Phosphatase Catalytic Subunit Dephosphorylates Cardiac Myosin via Mechanisms Dependent and Independent of the MYPT Regulatory Subunits,” Journal of Biological Chemistry 298 (2022): 102296. 35872014 10.1016/j.jbc.2022.102296 PMC9418503

[14] L. Naldini , U. Blömer , P. Gallay , et al., “In Vivo Gene Delivery and Stable Transduction of Nondividing Cells by a Lentiviral Vector,” Science 272 (1996): 263–267. 8602510 10.1126/science.272.5259.263

[15] S. J. Shankland , J. W. Pippin , J. Reiser , and P. Mundel , “Podocytes in culture: past, present, and future,” Kidney International 72 (2007): 26–36. 17457377 10.1038/sj.ki.5002291

[16] Y. Takano , K. Yamauchi , N. Hiramatsu , et al., “Recovery and Maintenance of Nephrin Expression in Cultured Podocytes and Identification of HGF as a Repressor of Nephrin,” American Journal of Physiology. Renal Physiology 292 (2007): F1573–F1582. 17244893 10.1152/ajprenal.00423.2006

[17] S. M. Heissler and D. J. Manstein , “Nonmuscle Myosin‐2: Mix and Match,” Cellular and Molecular Life Sciences 70 (2013): 1–21. 22565821 10.1007/s00018-012-1002-9 PMC3535348

[18] M. A. Conti and R. S. Adelstein , “Nonmuscle Myosin II Moves in New Directions,” Journal of Cell Science 121 (2008): 11–18. 18096687 10.1242/jcs.007112

[19] M. S. Shutova , S. B. Asokan , S. Talwar , R. K. Assoian , J. E. Bear , and T. M. Svitkina , “Self‐Sorting of Nonmuscle Myosins IIA and IIB Polarizes the Cytoskeleton and Modulates Cell Motility,” Journal of Cell Biology 216 (2017): 2877–2889. 28701425 10.1083/jcb.201705167 PMC5584186

[20] F. Kage , M. Vicente‐Manzanares , B. C. McEwan , A. N. Kettenbach , and H. N. Higgs , “Myosin II Proteins Are Required for Organization of Calcium‐Induced Actin Networks Upstream of Mitochondrial Division,” Molecular Biology of the Cell 33 (2022): ar63. 35427150 10.1091/mbc.E22-01-0005 PMC9561854

[21] Z. Liu , E. V. Rossen , J.‐P. Timmermans , A. Geerts , L. A. van Grunsven , and H. Reynaert , “Distinct Roles for Non‐Muscle Myosin II Isoforms in Mouse Hepatic Stellate Cells,” Journal of Hepatology 54 (2011): 132–141. 20932596 10.1016/j.jhep.2010.06.020

[22] E. Golomb , X. Ma , S. S. Jana , et al., “Identification and Characterization of Nonmuscle Myosin II‐C, a New Member of the Myosin II Family,” Journal of Biological Chemistry 279 (2004): 2800–2808. 14594953 10.1074/jbc.M309981200

[23] K. Yamada , “Optimization of Allosteric With‐No‐Lysine (WNK) Kinase Inhibitors and Efficacy in Rodent Hypertension Models,” Journal of Medicinal Chemistry 60 (2017): 7099–7107. 28771350 10.1021/acs.jmedchem.7b00708

[24] S. Jiang , F. Alisafaei , Y. Y. Huang , et al., “An Ex Vivo Culture Model of Kidney Podocyte Injury Reveals Mechanosensitive, Synaptopodin‐Templating, Sarcomere‐Like Structures,” Science Advances 8 (2022): eabn6027. 36044576 10.1126/sciadv.abn6027 PMC9432837

[25] H. Y. Suleiman , R. Roth , S. Jain , J. E. Heuser , A. S. Shaw , and J. H. Miner , “Injury‐Induced Actin Cytoskeleton Reorganization in Podocytes Revealed by Super‐Resolution Microscopy,” JCI Insight 2 (2017). 10.1172/jci.insight.94137 PMC5621879 28814668

[26] J. Yoon , Z. Liu , M. Alaba , et al., “Glomerular Elasticity and Gene Expression Patterns Define Two Phases of Alport Nephropathy,” bioRxiv (2024).

[27] K. L. Andrews , J. L. Mudd , C. Li , and J. H. Miner , “Quantitative Trait Loci Influence Renal Disease Progression in a Mouse Model of Alport Syndrome,” American Journal of Pathology 160 (2002): 721–730. 11839593 10.1016/S0002-9440(10)64892-4 PMC1850644

[28] K. E. Kamm and J. T. Stull , “Signaling to Myosin Regulatory Light Chain in Sarcomeres,” Journal of Biological Chemistry 286 (2011): 9941–9947. 21257758 10.1074/jbc.R110.198697 PMC3060548

[29] E. K. Blue , Z. M. Goeckeler , Y. Jin , et al., “220‐ and 130‐kDa MLCKs Have Distinct Tissue Distributions and Intracellular Localization Patterns,” American Journal of Physiology. Cell Physiology 282 (2002): C451–C460. 11832329 10.1152/ajpcell.00333.2001 PMC2823798

[30] M. S. Wainwright , J. Rossi , J. Schavocky , et al., “Protein Kinase Involved in Lung Injury Susceptibility: Evidence From Enzyme Isoform Genetic Knockout and in Vivo Inhibitor Treatment,” Proceedings of the National Academy of Sciences of the United States of America 100 (2003): 6233–6238. 12730364 10.1073/pnas.1031595100 PMC156355

[31] A. P. Somlyo and A. V. Somlyo , “Ca2+ Sensitivity of Smooth Muscle and Nonmuscle Myosin II: Modulated by G Proteins, Kinases, and Myosin Phosphatase,” Physiological Reviews 83 (2003): 1325–1358. 14506307 10.1152/physrev.00023.2003

[32] M. Ikebe , D. J. Hartshorne , and M. Elzinga , “Identification, Phosphorylation, and Dephosphorylation of a Second Site for Myosin Light Chain Kinase on the 20,000‐Dalton Light Chain of Smooth Muscle Myosin,” Journal of Biological Chemistry 261 (1986): 36–39. 3079756

[33] L. Peng , J. Ma , R. Cui , et al., “The Calcineurin Inhibitor Tacrolimus Reduces Proteinuria in Membranous Nephropathy Accompanied by a Decrease in Angiopoietin‐Like‐4,” PLoS One 9 (2014): e106164. 25165975 10.1371/journal.pone.0106164 PMC4148427

[34] M. Wang , J. Zhou , Q. Niu , and H. Wang , “Mechanism of Tacrolimus in the Treatment of Lupus Nephritis,” Frontiers in Pharmacology 15 (2024). 10.3389/fphar.2024.1331800 PMC11106426 38774214

[35] X. Li , H. Li , H. Ye , et al., “Tacrolimus Therapy in Adults With Steroid‐ and Cyclophosphamide‐Resistant Nephrotic Syndrome and Normal or Mildly Reduced GFR,” American Journal of Kidney Diseases 54 (2009): 51–58. 19406543 10.1053/j.ajkd.2009.02.018

[36] F. C. Branco and P. Camurça Fernandes , “Treatment of Steroid‐Resistant Nephrotic Syndrome With Cyclosporine: Study of 17 Cases and a Literature Review,” Journal of Nephrology 18 (2005): 711–720. 16358229

[37] C. J. Stefanidis and U. Querfeld , “The Podocyte as a Target: Cyclosporin A in the Management of the Nephrotic Syndrome Caused by WT1 Mutations,” European Journal of Pediatrics 170 (2011): 1377–1383. 21298518 10.1007/s00431-011-1397-6

[38] J. Hogan and J. Radhakrishnan , “The Treatment of Minimal Change Disease in Adults,” J Am Soc Nephrol 24 (2013): 702–711. 23431071 10.1681/ASN.2012070734

[39] Y. Peleg , A. S. Bomback , and J. Radhakrishnan , “The Evolving Role of Calcineurin Inhibitors in Treating Lupus Nephritis,” Clinical Journal of the American Society of Nephrology 15 (2020): 1066–1072. 32152065 10.2215/CJN.13761119 PMC7341791

[40] R. Liao , Q. Liu , Z. Zheng , et al., “Tacrolimus Protects Podocytes From Injury in Lupus Nephritis Partly by Stabilizing the Cytoskeleton and Inhibiting Podocyte Apoptosis,” PLoS One 10 (2015): e0132724. 26161538 10.1371/journal.pone.0132724 PMC4498640

[41] X. Shen , H. Jiang , M. Ying , et al., “Calcineurin Inhibitors Cyclosporin A and Tacrolimus Protect Against Podocyte Injury Induced by Puromycin Aminonucleoside in Rodent Models,” Scientific Reports 6 (2016): 32087. 27580845 10.1038/srep32087 PMC5007516

[42] P. Mundel , J. Reiser , A. Zúñiga Mejía Borja , et al., “Rearrangements of the Cytoskeleton and Cell Contacts Induce Process Formation During Differentiation of Conditionally Immortalized Mouse Podocyte Cell Lines,” Experimental Cell Research 236 (1997): 248–258. 9344605 10.1006/excr.1997.3739

[43] J. Oh , J. Reiser , and P. Mundel , “Dynamic (Re)organization of the Podocyte Actin Cytoskeleton in the Nephrotic Syndrome,” Pediatric Nephrology 19 (2004): 130–137. 14673634 10.1007/s00467-003-1367-y

[44] A. Poperechnaya , O. Varlamova , P. J. Lin , J. T. Stull , and A. R. Bresnick , “Localization and Activity of Myosin Light Chain Kinase Isoforms During the Cell Cycle,” Journal of Cell Biology 151 (2000): 697–708. 11062269 10.1083/jcb.151.3.697 PMC2185581

[45] L. S. Velasquez , L. B. Sutherland , Z. Liu , et al., “Activation of MRTF‐A‐Dependent Gene Expression With a Small Molecule Promotes Myofibroblast Differentiation and Wound Healing,” Proceedings of the National Academy of Sciences of the United States of America 110 (2013): 16850–16855. 24082095 10.1073/pnas.1316764110 PMC3801009

[46] X. Fan , H. Yang , S. Kumar , et al., “SLIT2/ROBO2 Signaling Pathway Inhibits Nonmuscle Myosin IIA Activity and Destabilizes Kidney Podocyte Adhesion,” JCI Insight 1 (2016): e86934. 27882344 10.1172/jci.insight.86934 PMC5111509

[47] C. Schell , M. Rogg , M. Suhm , et al., “The FERM Protein EPB41L5 Regulates Actomyosin Contractility and Focal Adhesion Formation to Maintain the Kidney Filtration Barrier,” Proceedings of the National Academy of Sciences of the United States of America 114 (2017): E4621–E4630. 28536193 10.1073/pnas.1617004114 PMC5468651

[48] P. Rachubik , “Insulin Controls Cytoskeleton Reorganization and Filtration Barrier Permeability via the PKGIalpha‐Rac1‐RhoA Crosstalk in Cultured Rat Podocytes,” Biochimica et Biophysica Acta, Molecular Cell Research 1869 (2022): 119301. 35642843 10.1016/j.bbamcr.2022.119301

[49] E. Kassianidou , J. H. Hughes , and S. Kumar , “Activation of ROCK and MLCK Tunes Regional Stress Fiber Formation and Mechanics via Preferential Myosin Light Chain Phosphorylation,” Molecular Biology of the Cell 28 (2017): 3832–3843. 29046396 10.1091/mbc.E17-06-0401 PMC5739298

[50] J. U. Jung , S. Stippec , and M. H. Cobb , “Activation of WNK1 Signaling Through Piezo1,” Proceedings of the National Academy of Sciences of the United States of America 122 (2025): e2513155122. 40880539 10.1073/pnas.2513155122 PMC12415193

[51] D. B. Johnstone , J. Zhang , B. George , et al., “Podocyte‐Specific Deletion of Myh9 Encoding Nonmuscle Myosin Heavy Chain 2A Predisposes Mice to Glomerulopathy,” Molecular and Cellular Biology 31 (2011): 2162–2170. 21402784 10.1128/MCB.05234-11 PMC3133349

