# Supplementary material for: WNK1 Kinase Activity Is Required for the Functional Maintenance of Podocyte Structure
Source: FASEB J. 2026 Feb 11;40(4):e71551. doi: 10.1096/fj.202503839R (PMC12892244; doi:10.1096/fj.202503839R)
Supplement: Supplementary file 1 — Data S1: Supplementary Figures. [file FSB2-40-e71551-s001.docx]

**Supplementary Material**

|  | **B.**  **** |
| --- | --- |

| **C.**  **** |
| --- |

**Figure S1. Quantification of glomerular podocytes. A)** To assess effects of WNK463 on glomerular volume, post-WNK463 treatment via oral gavage, cryosections of renal cortices were stained for WT-1 immunofluorescence, and the podocyte density was quantified, using the method published by the Wiggan’s group (Venkatareddy, Madhusudan et al. “Estimating podocyte number and density using a single histologic section.” *Journal of the American Society of Nephrology : JASN* vol. 25,5 (2014): 1118-29. doi:10.1681/ASN.2013080859). Podocyte density is tightly associated with glomerular volume. **B)** Comparison showed no significant difference in podocyte density between Control (N=22 glomeruli) and WNK463 (N=34 glomeruli) treated groups. C) Quantification of total podocyte slit diaphragm markers nephrin and synaptopodin in vehicle control and WNK463 treated kidneys, determined by immunoblots of total kidney homogenates after separation by 4-12% SDS-PAGE. β-actin was used as loading control. Despite reduction in synaptopodin by immunofluorescence, total protein expression is unchanged with WNK463 treatment.

**Figure S2. Comparison of NMII paralog expression in WT podocyte cell line and primary podocytes isolated from WT mice.**  Podocyte cell lysates were quantified by Bradford assay and equivalent amounts loaded next to protein standards in the same gel to quantify molar ratios of specific paralog expression. For NMIIA and NMIIB, 1.5 ug or total protein were loaded. For NMIIC, 4.5 ug of total protein was loaded. Purified NMIIs were loaded from 20 to 80 fmol for NMIIA, NMIIB, and 7 to 42 fmol for NMIIC. Protein standards vary in size due to presence or absence of Halo tag. βactin was used as loading control for the podocyte cell lysates.

**A.**

| **NMIIA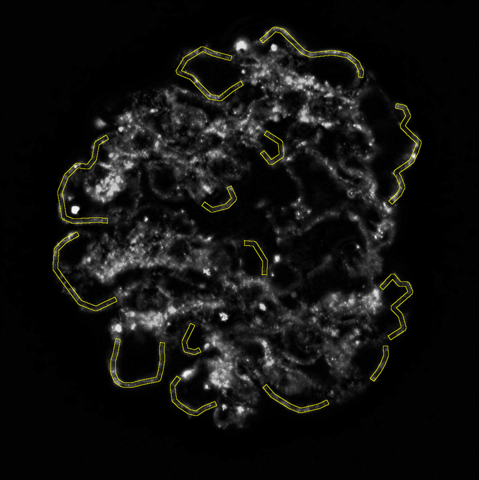** | **NMIIB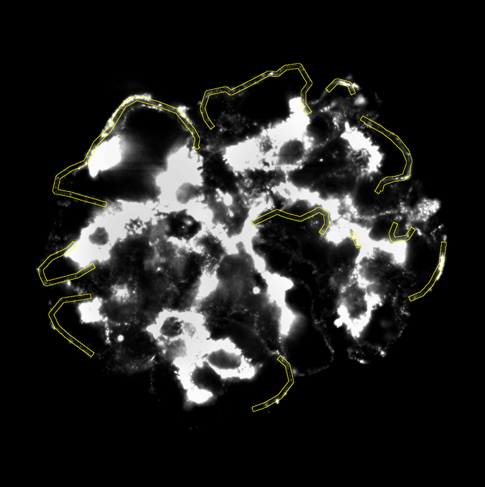** | **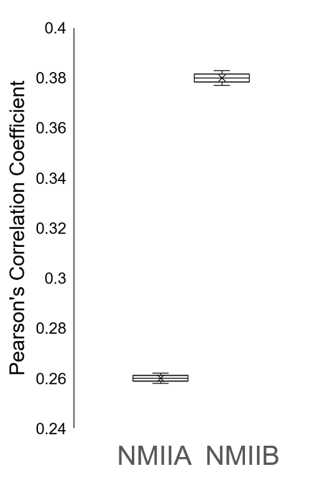** |
| --- | --- | --- |

**B.**

**Figure S3. Quantification of IF images.** A) Pearson’s correlation coefficient of NMIIA and NMIIB with synaptopodin-positive outer edges of capillary loops. B) Total RLC-P signal relative to phalloidin-stain quantified from total number of individual cells indicated within bar. RLC-P signal is significantly increased in FK506-treated cells, and significantly decreased compared to serum-free (SF) conditions.

| **A.**  **** | **B.**  **** |
| --- | --- |

**Figure S4. Confirmation of drug efficacy.** Reagents used for *in vivo* and *ex vivo* treatments of mice and isolated glomeruli were tested using WT podocytes in culture. A) Representative immunofluorescence images show enhanced pSPAK/OSR1 signal with FK506 treatment, that is reduced with WNK inhibitor WNK463. B) Representative immunoblots of podocyte cell lysates after treatments indicated, shows consistent signal pattern. NMII regulatory light chain phosphorylation (RLC-P) also showed similar pattern of activation with FK506 and inhibition with WNK463.

**
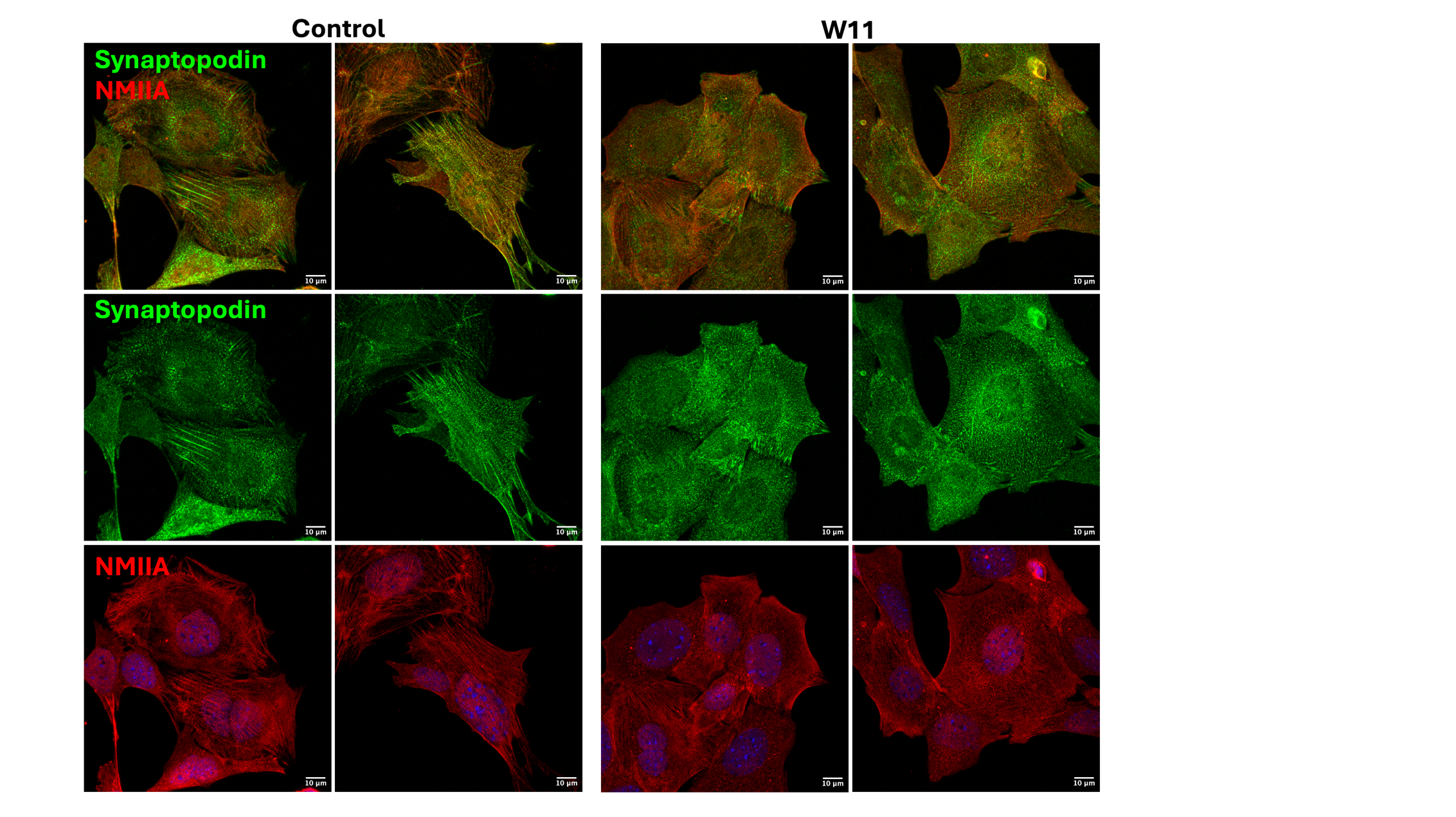
**

**Figure S5. Effect of W11 on the distribution of synaptopodin and NMIIA in VRAD-differentiated podocytes with sarcomere-like structures.**

**
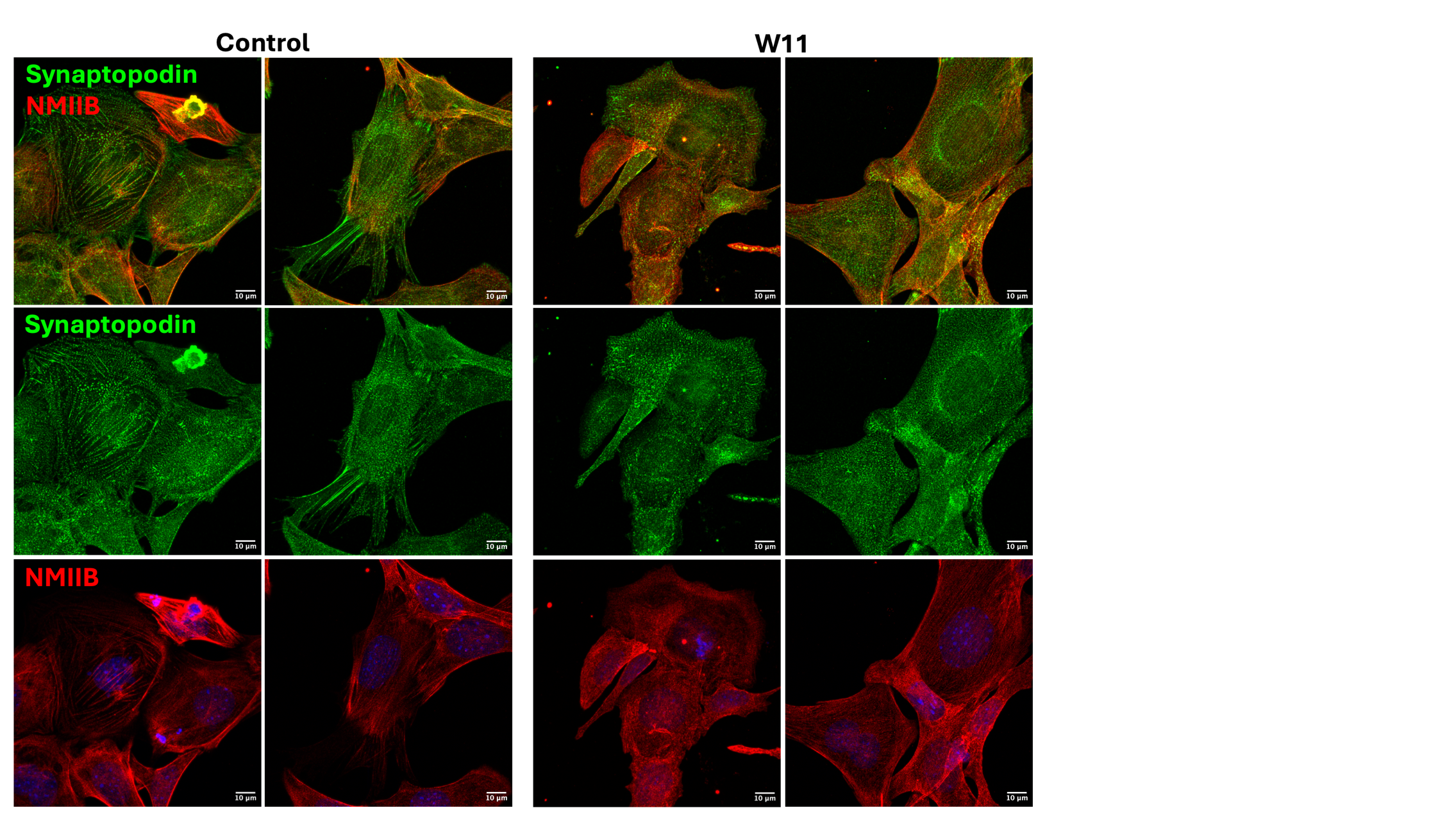
**

**Figure S6. Effect of W11 on the distribution of synaptopodin and NMIIA in VRAD-differentiated podocytes with sarcomere-like structures.**

**A.**

| 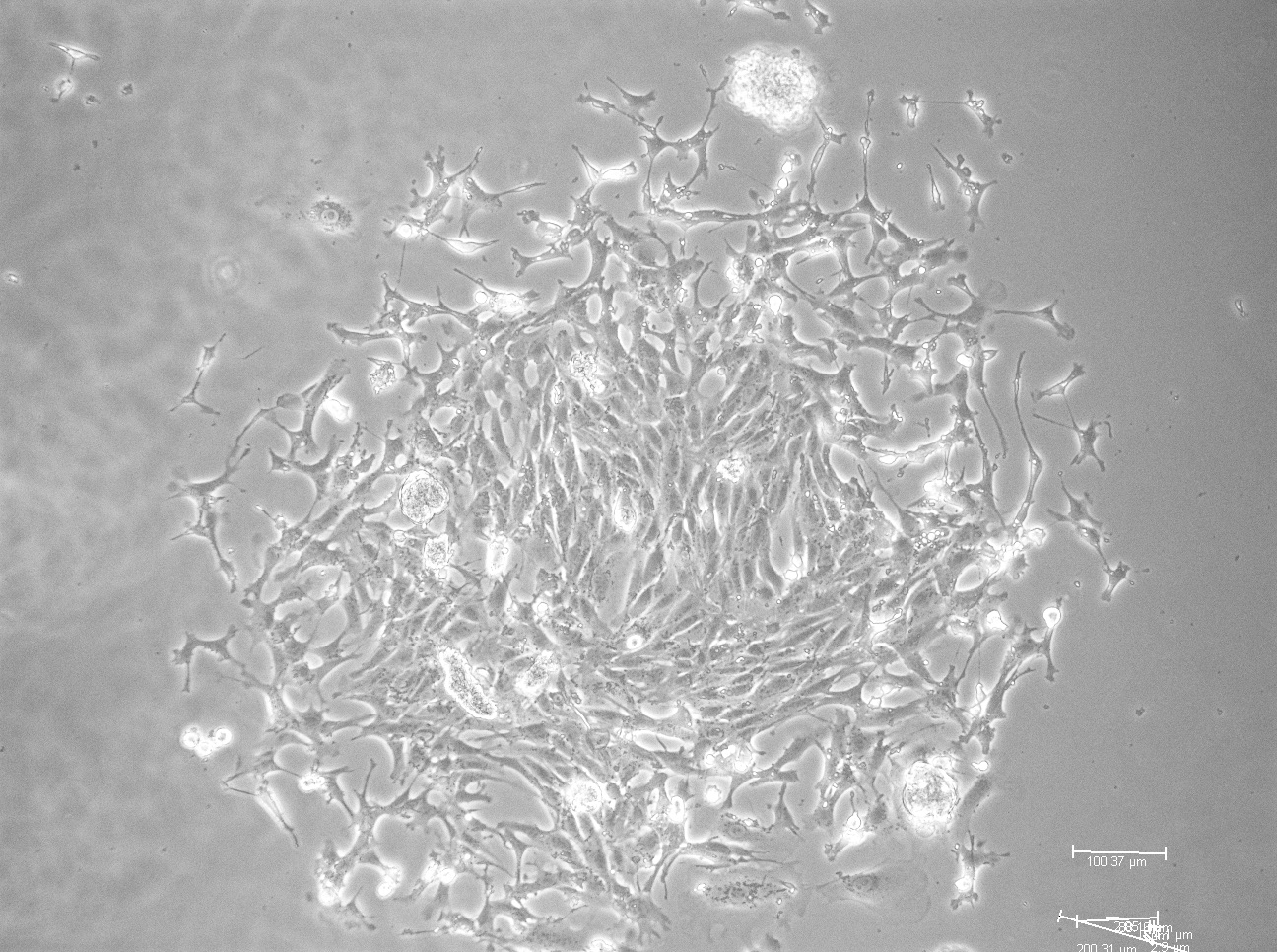 | 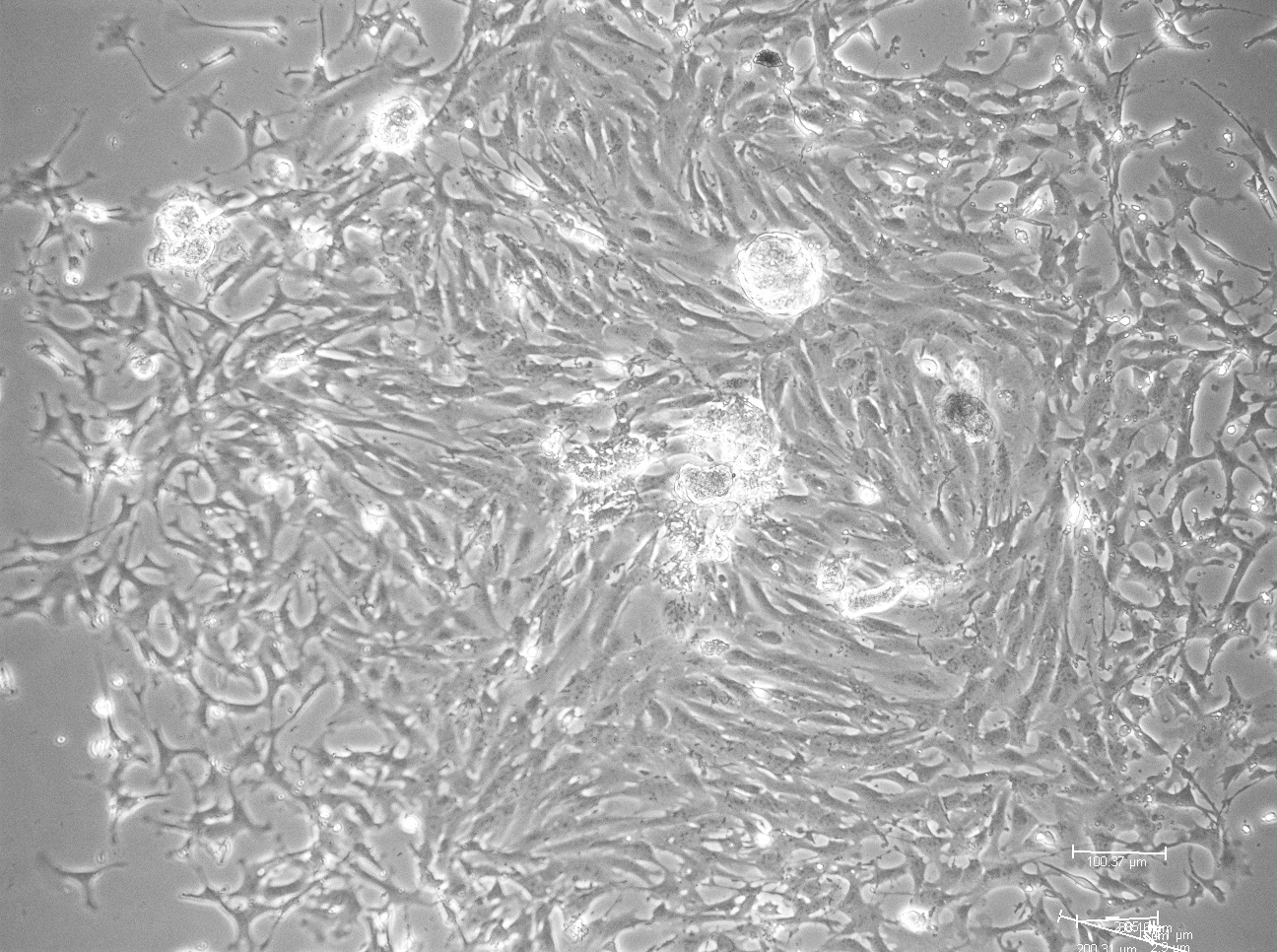 | 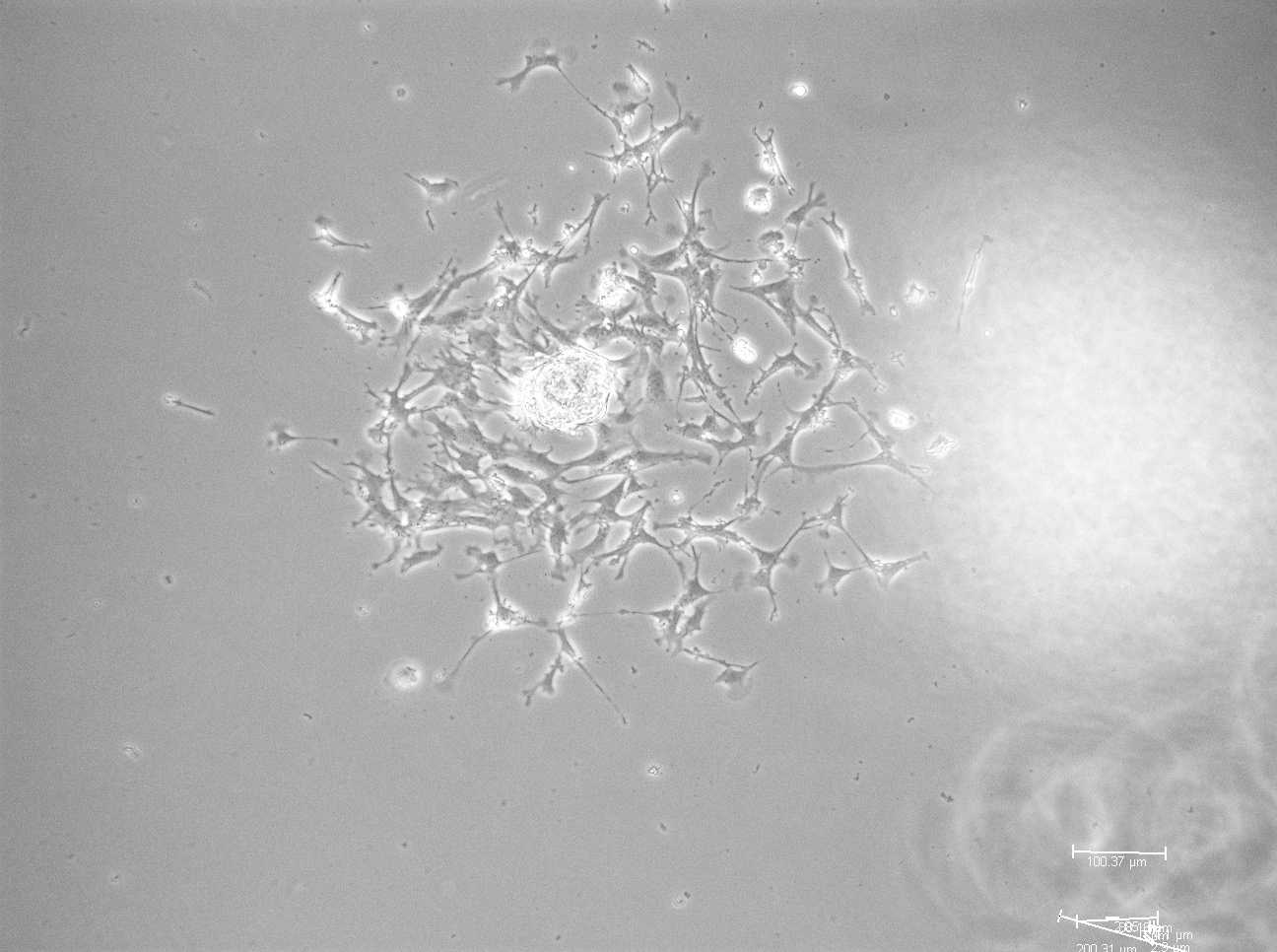 | 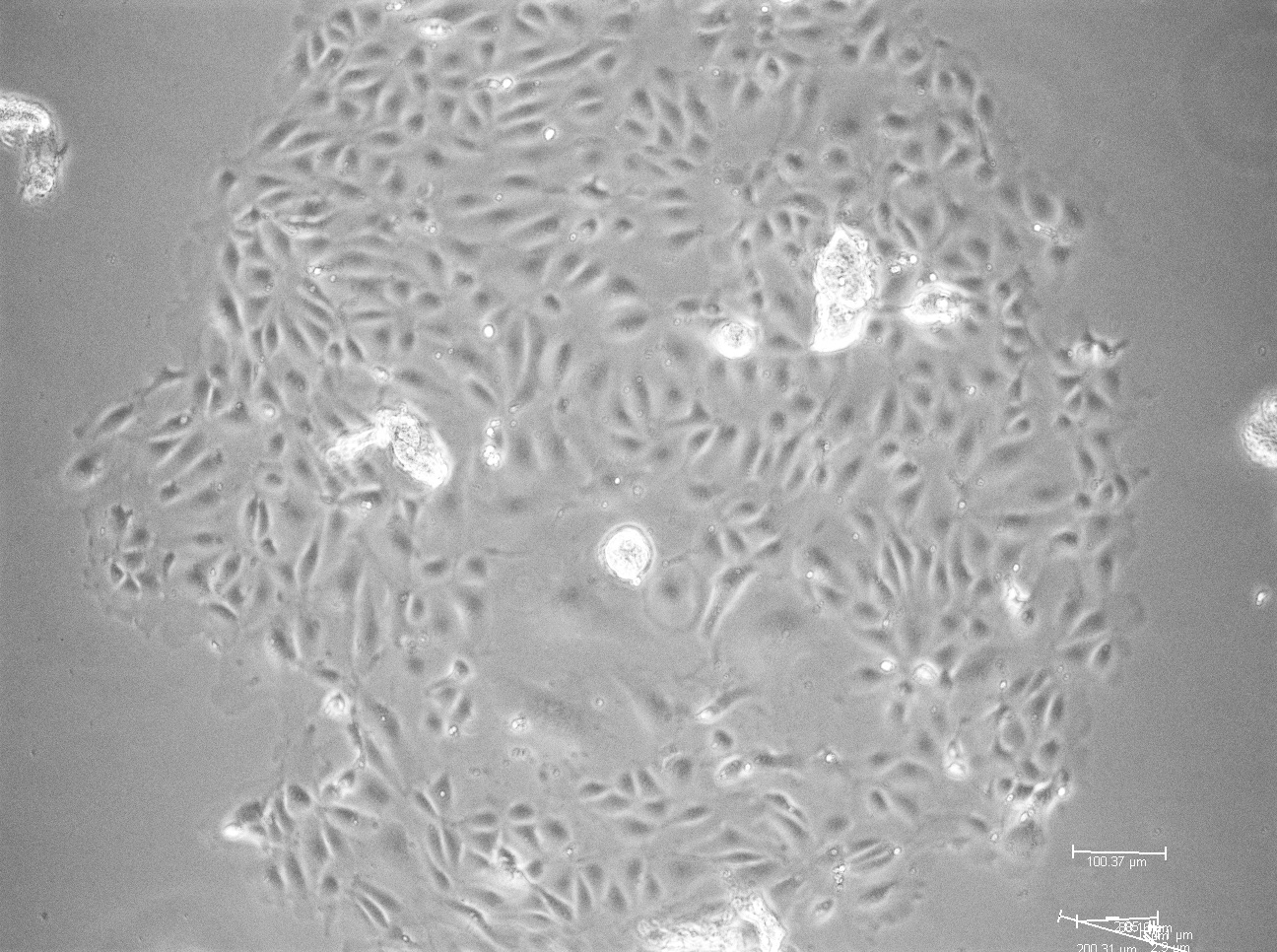 |
| --- | --- | --- | --- |
| 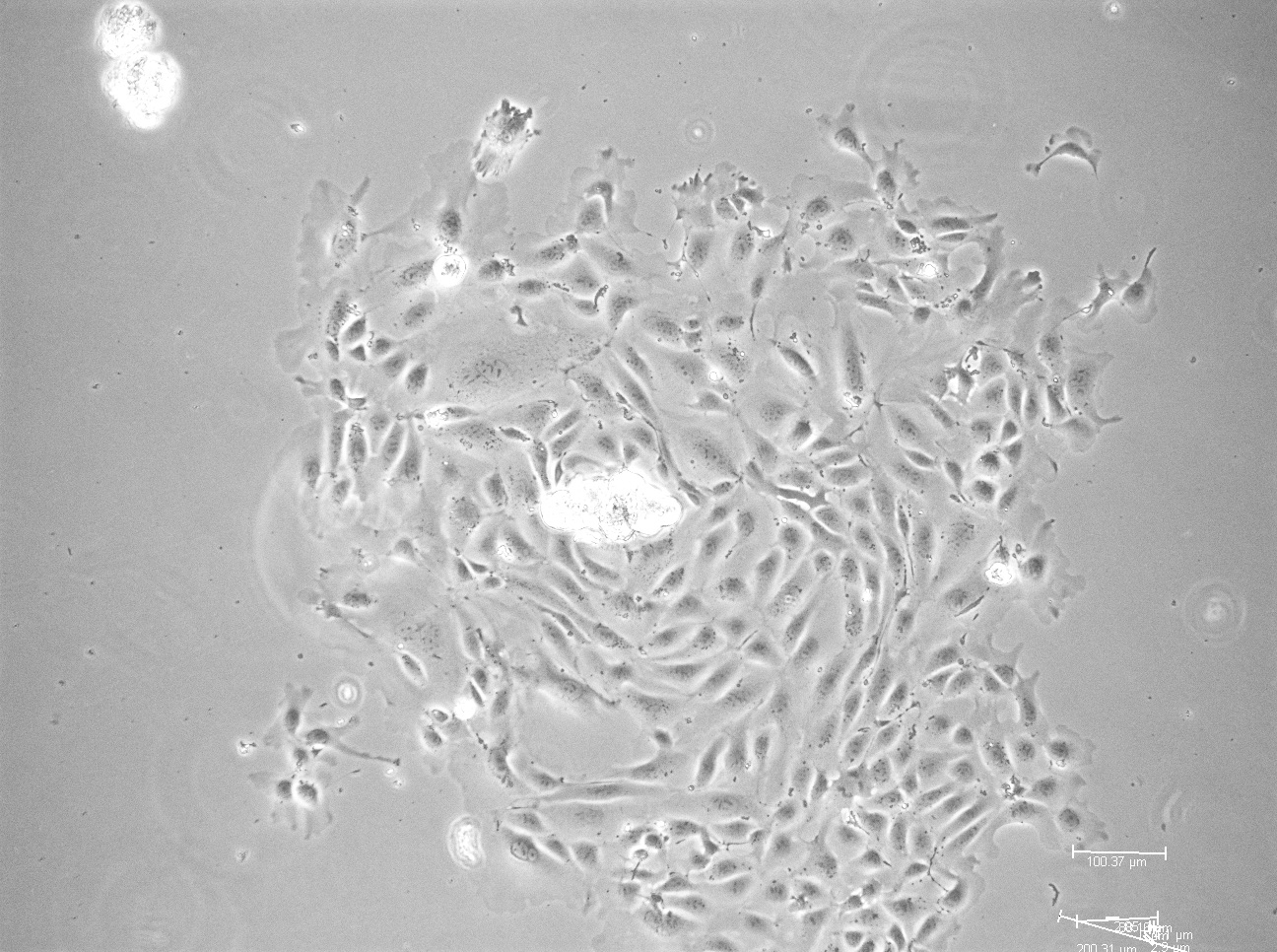 | 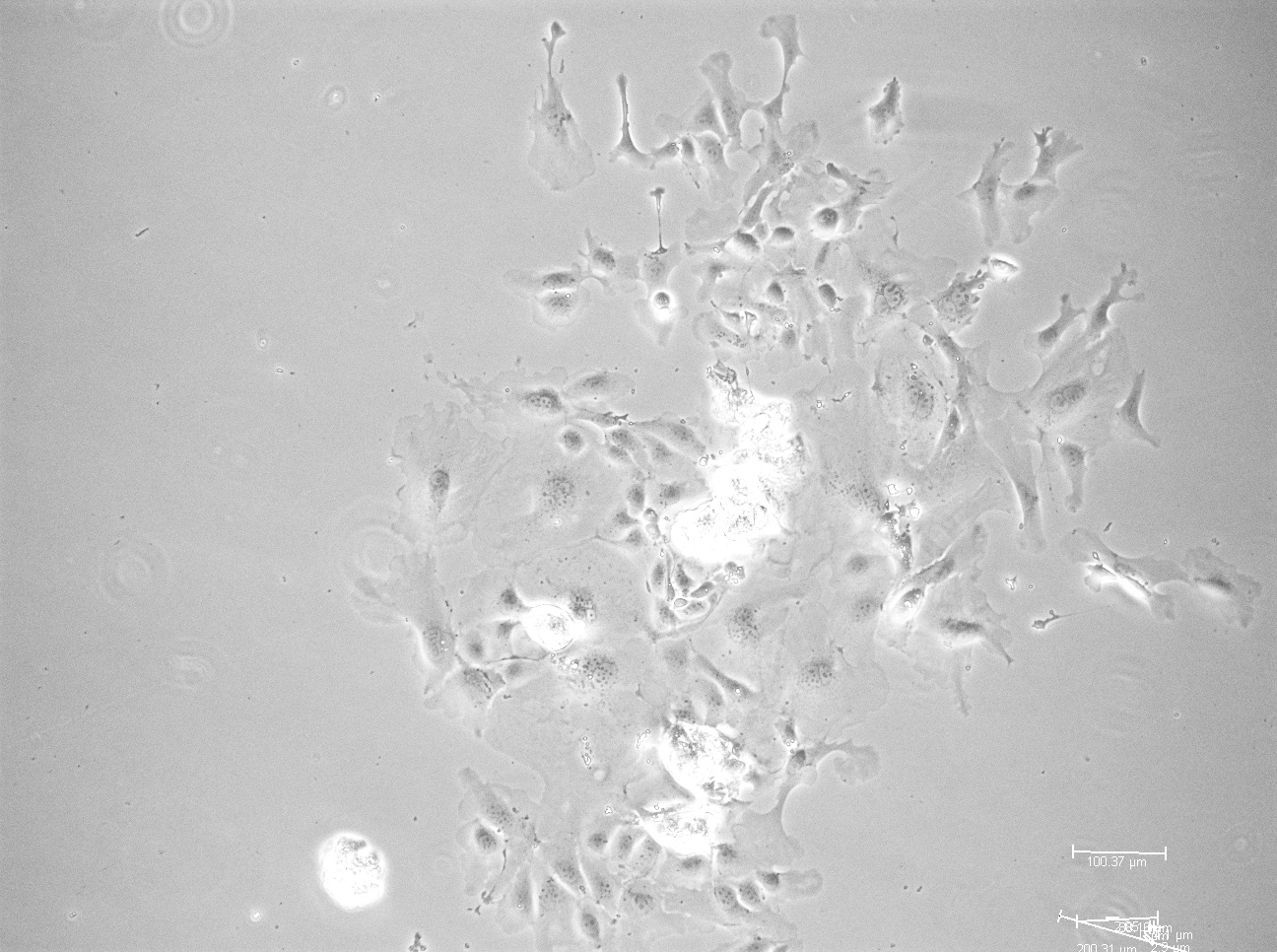 | 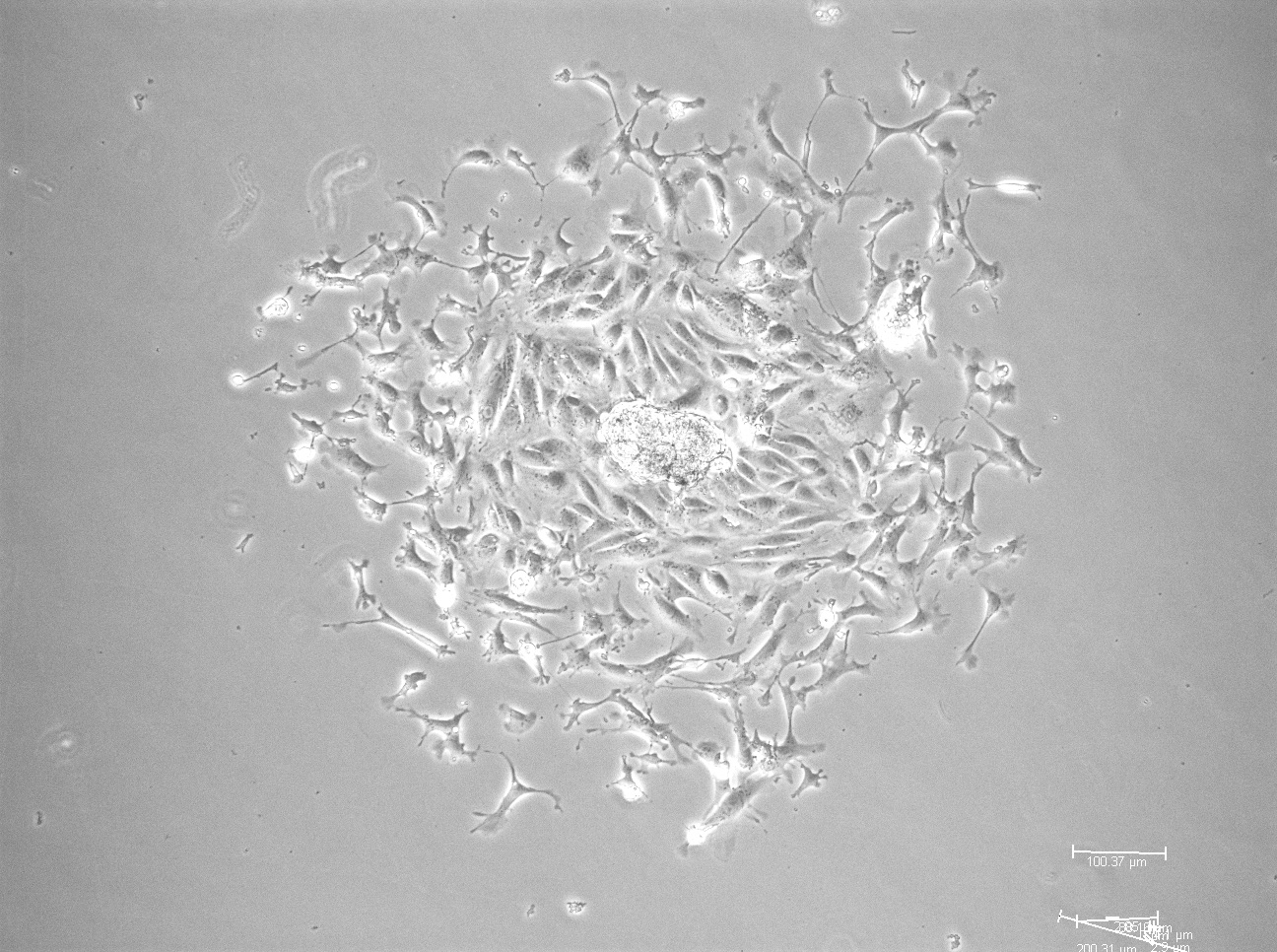 | 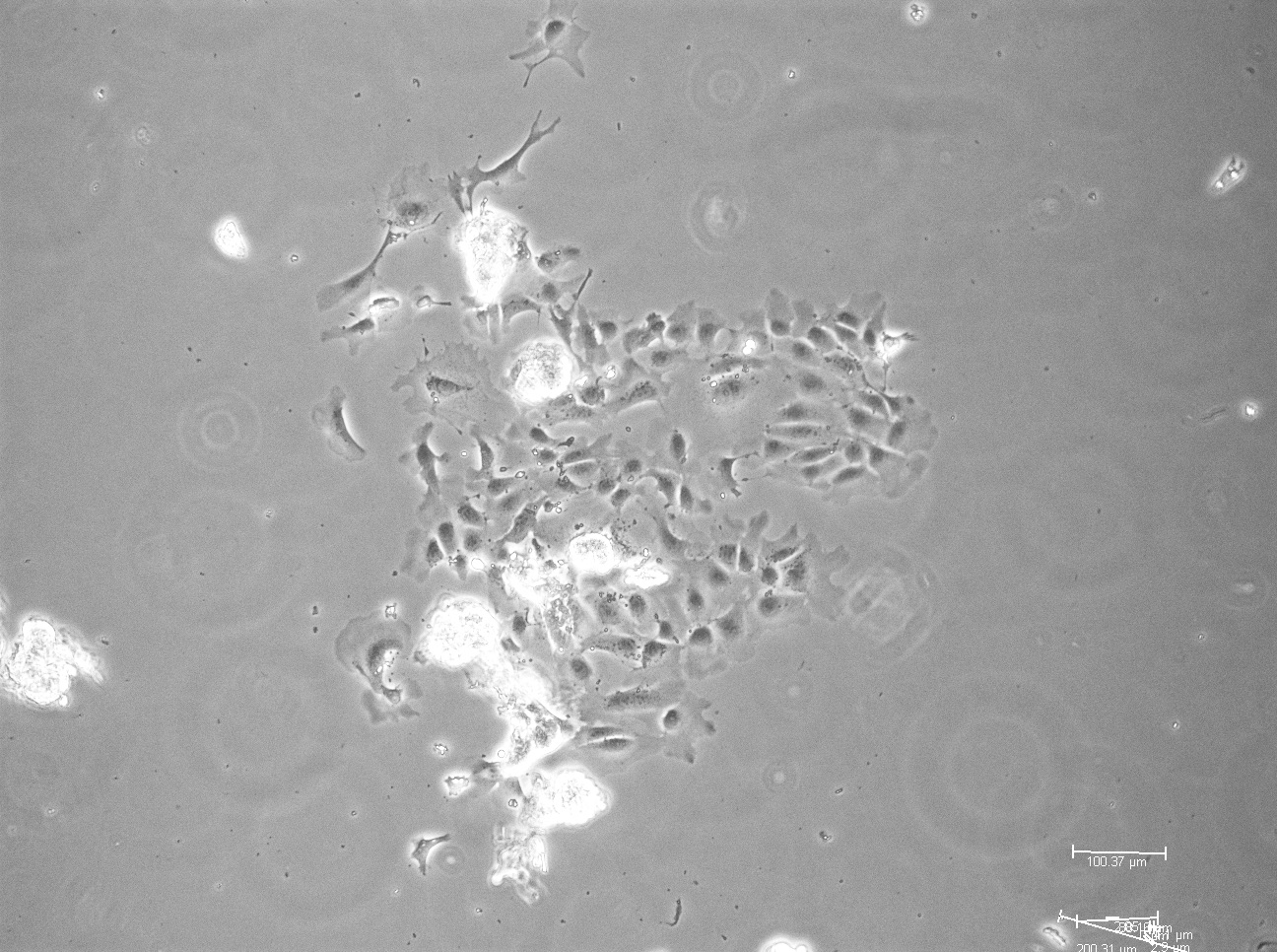 |
| 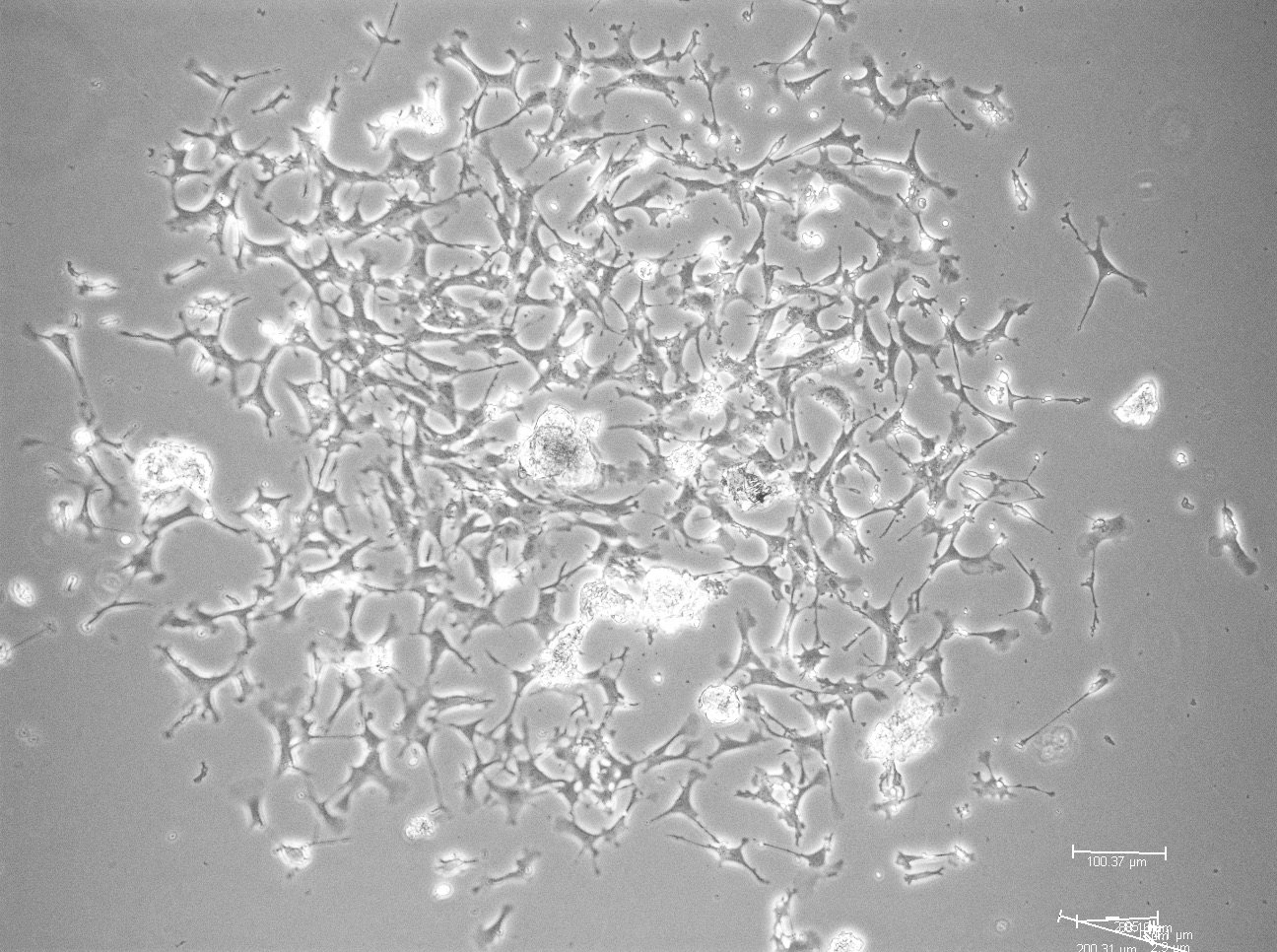 | 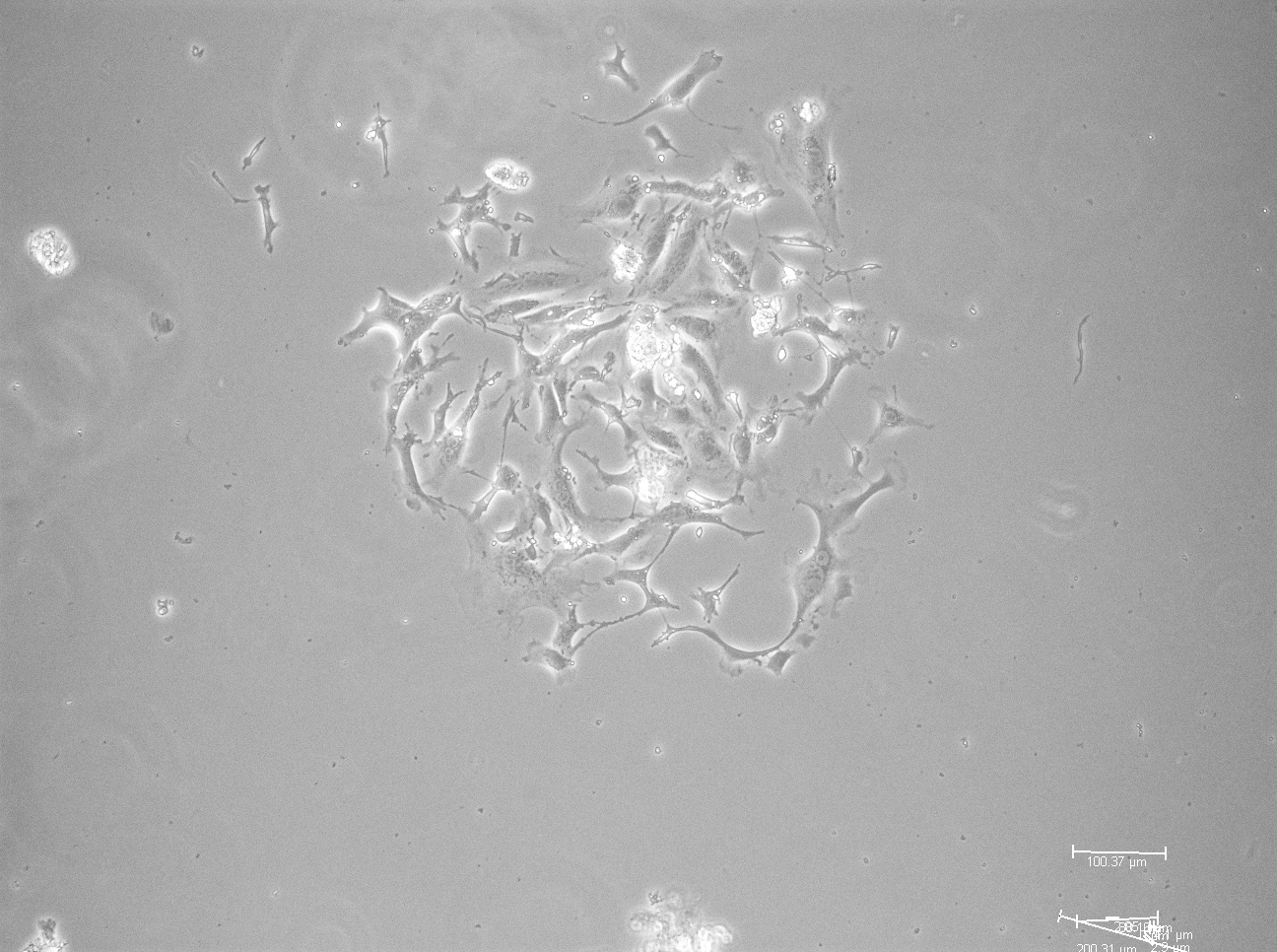 | 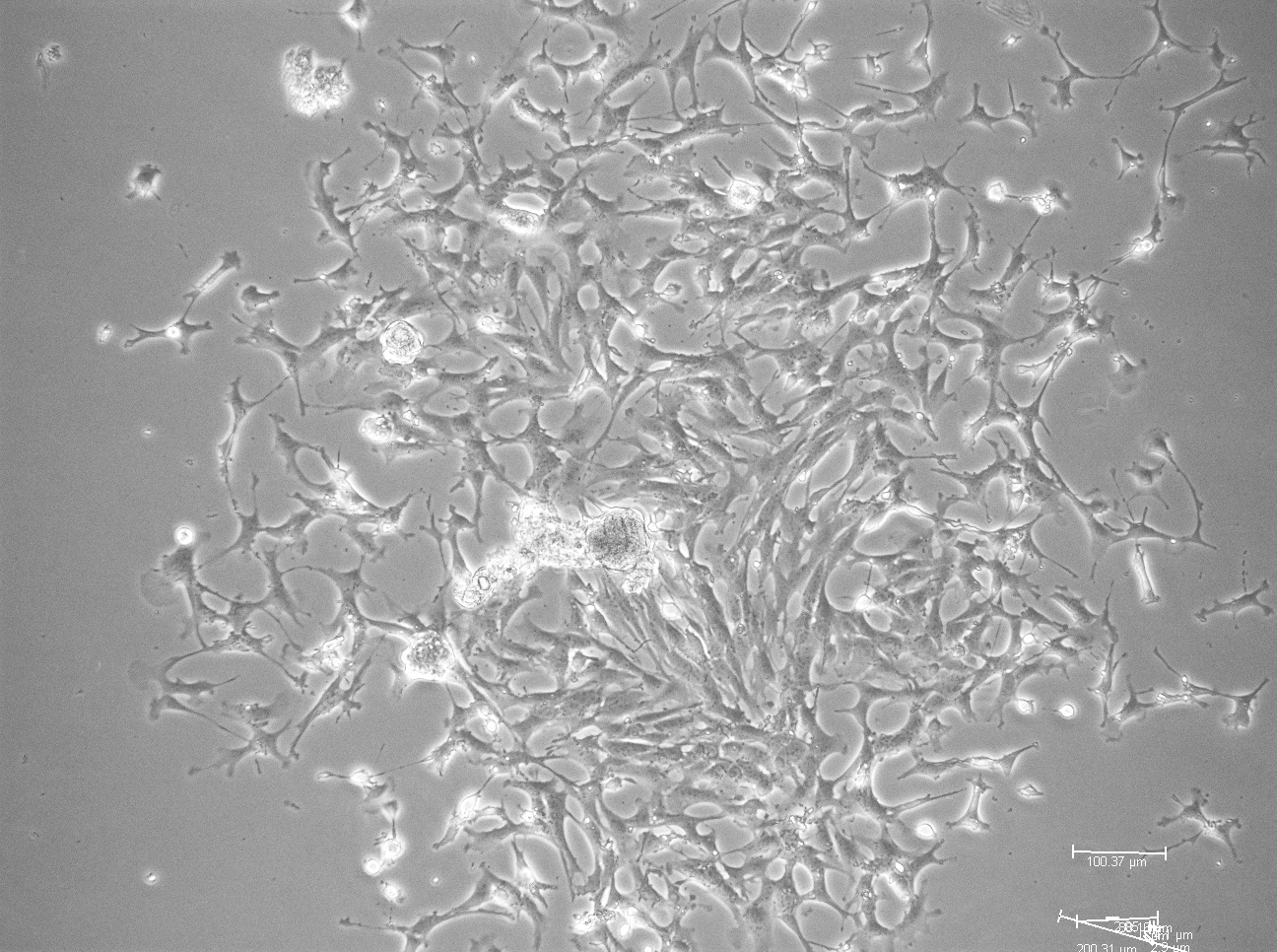 | 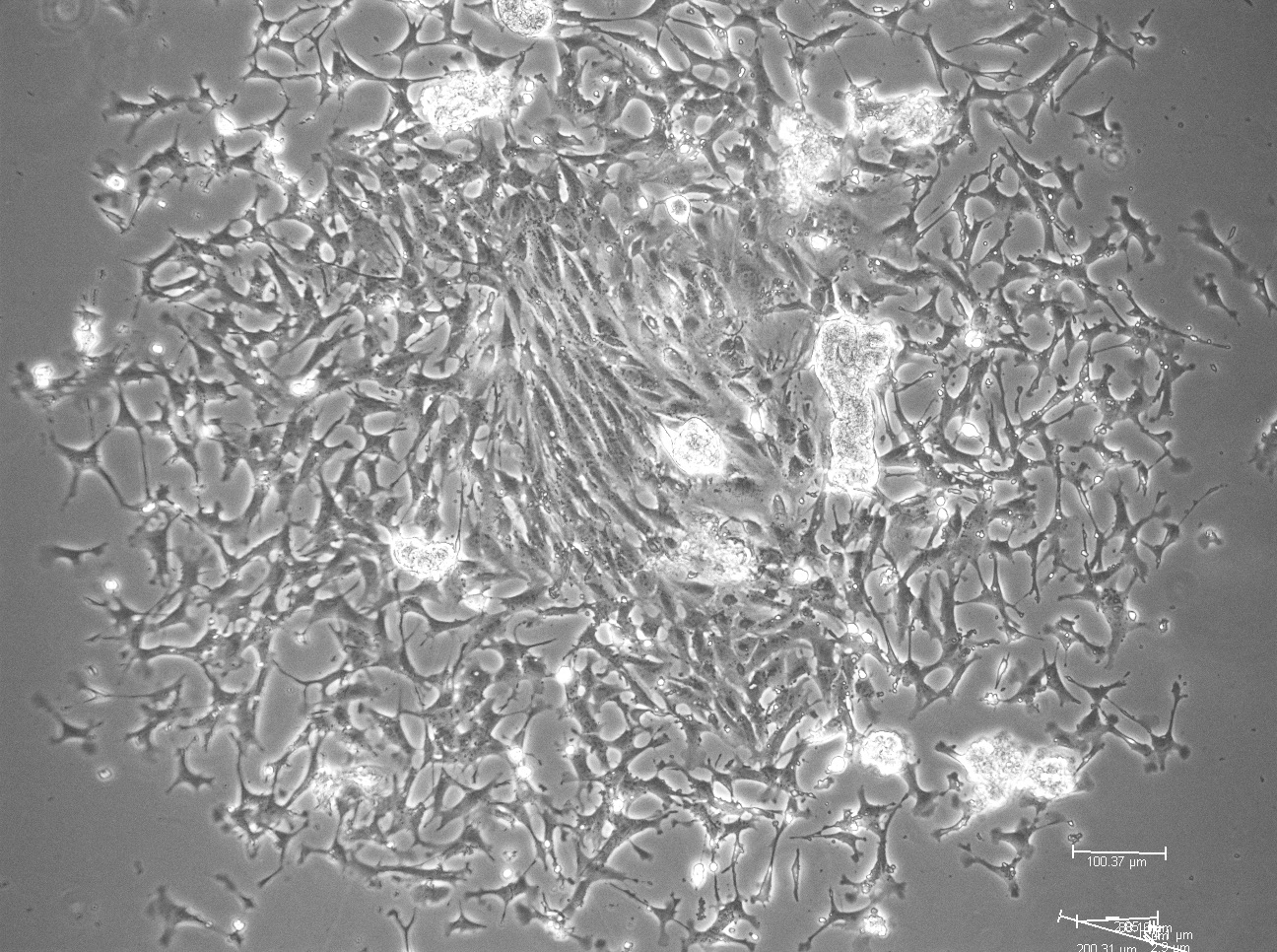 |

**B.**

| 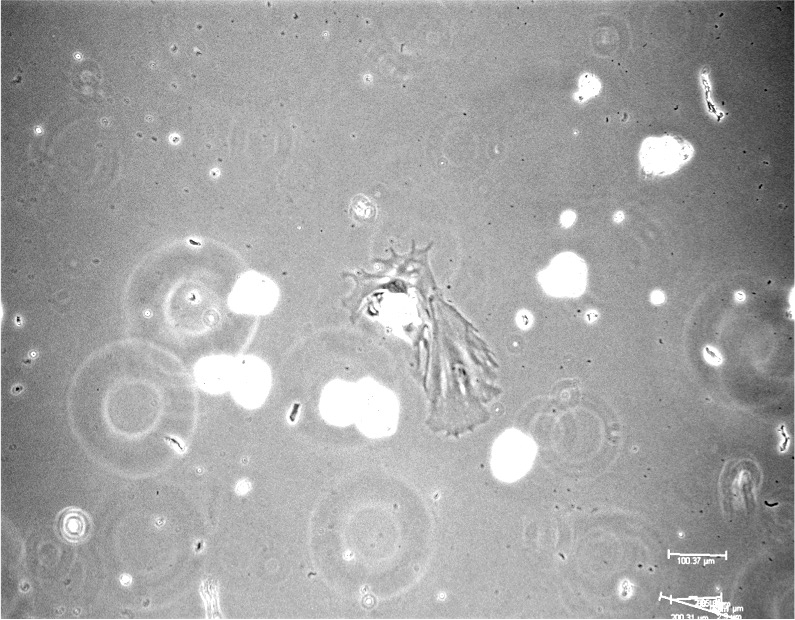 | 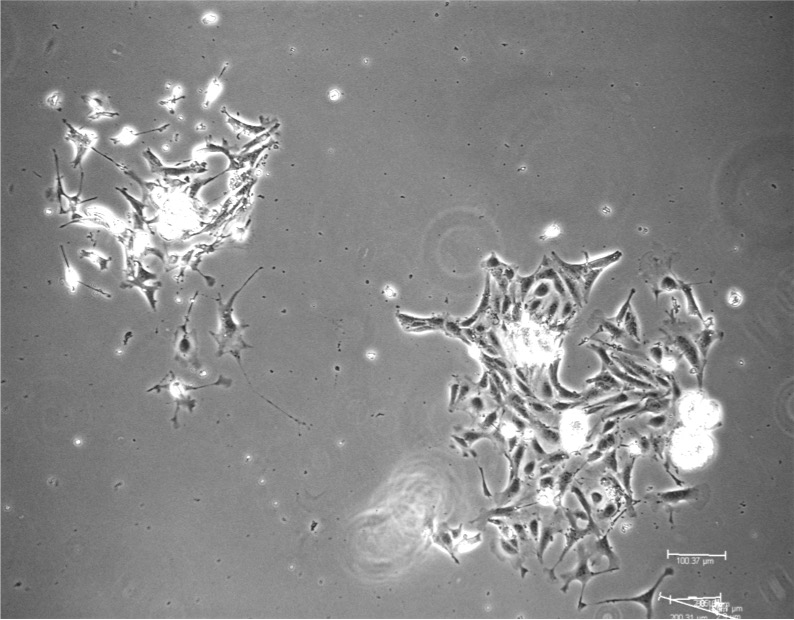 | 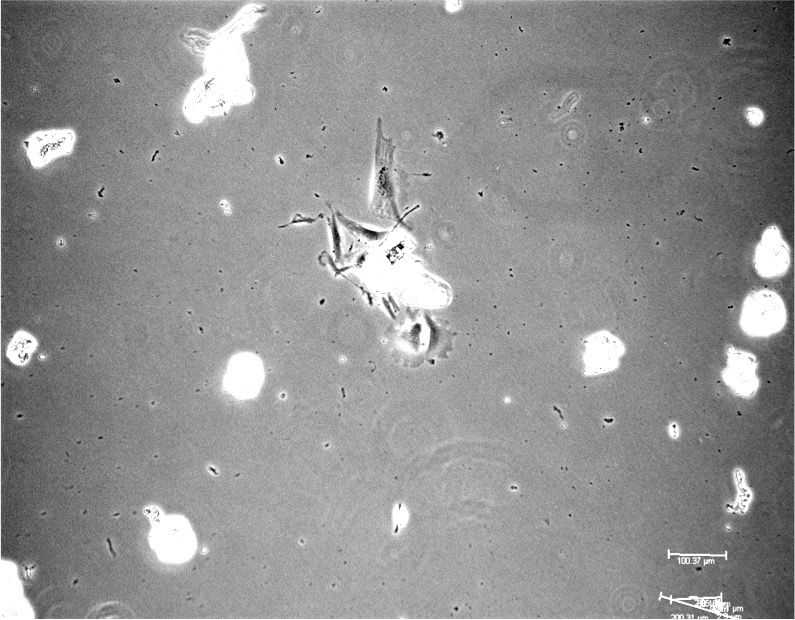 | 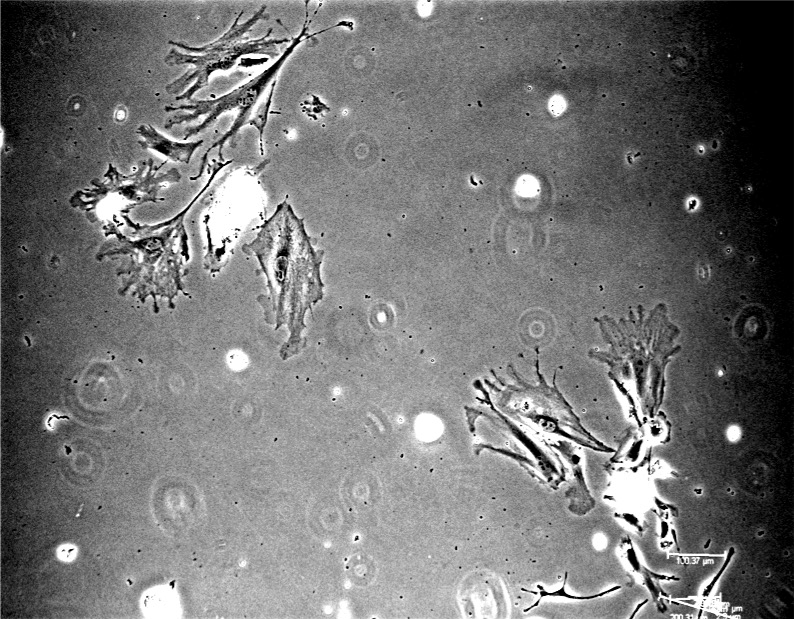 |
| --- | --- | --- | --- |
| 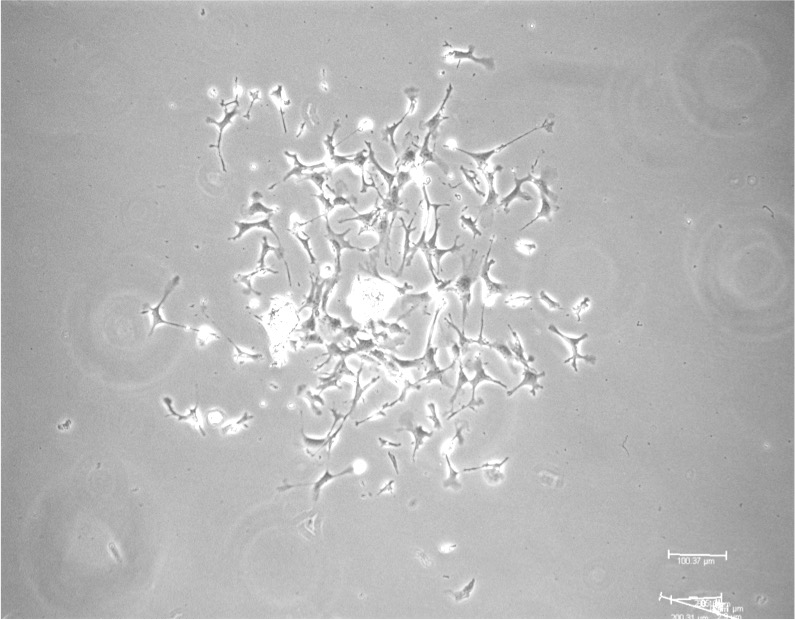 | 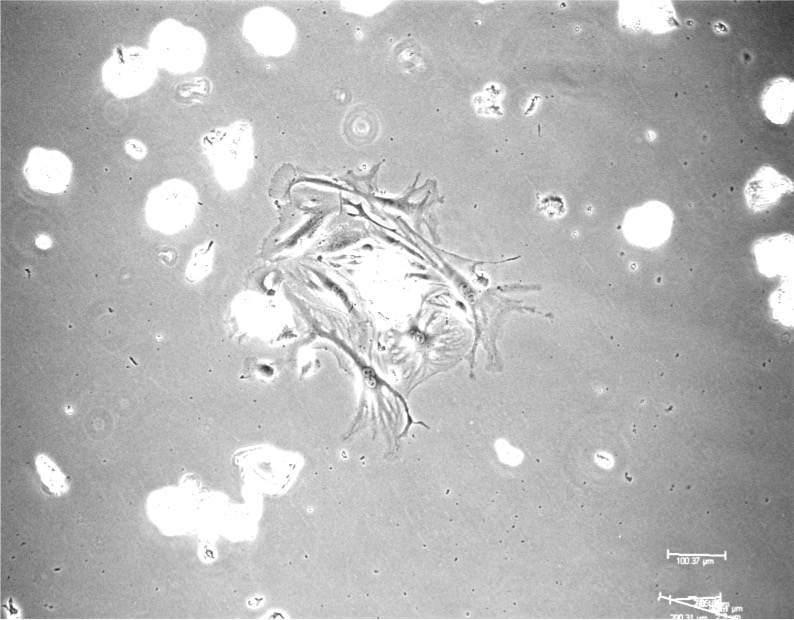 | 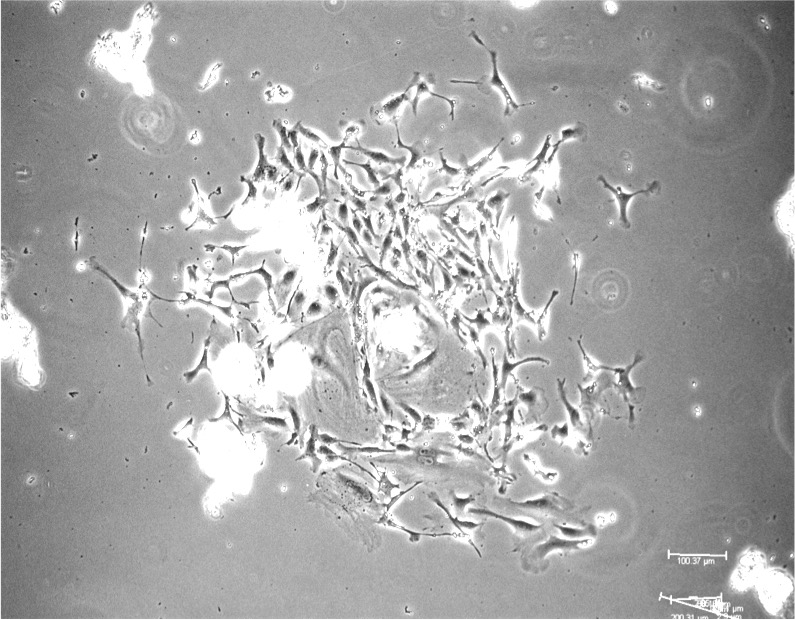 | 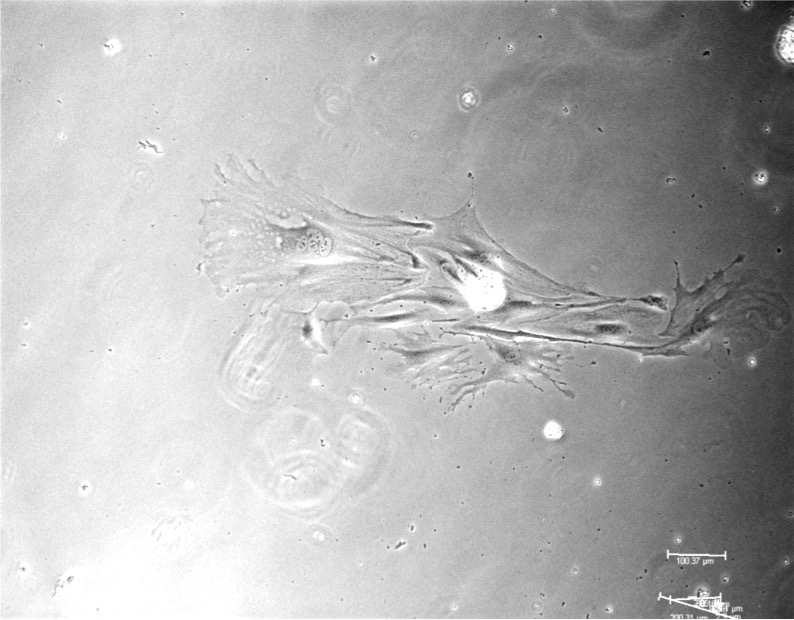 |
| 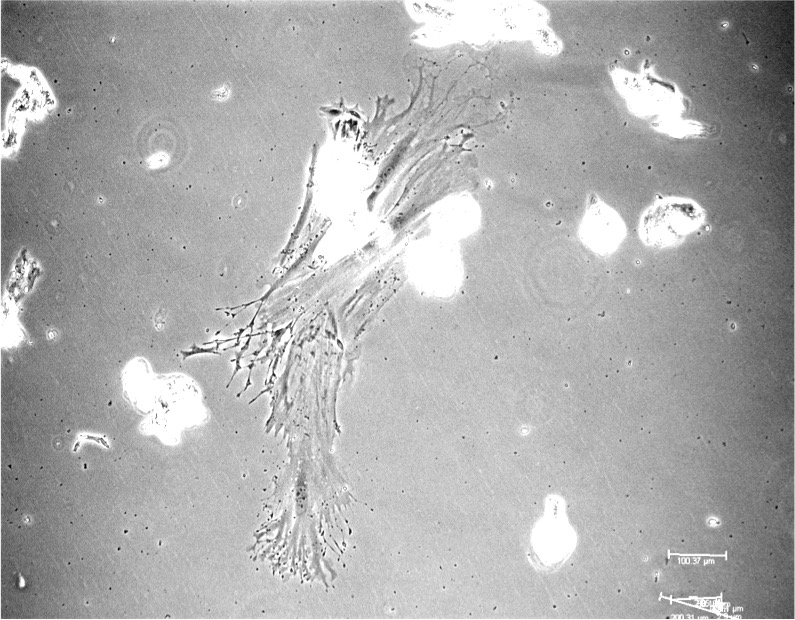 | 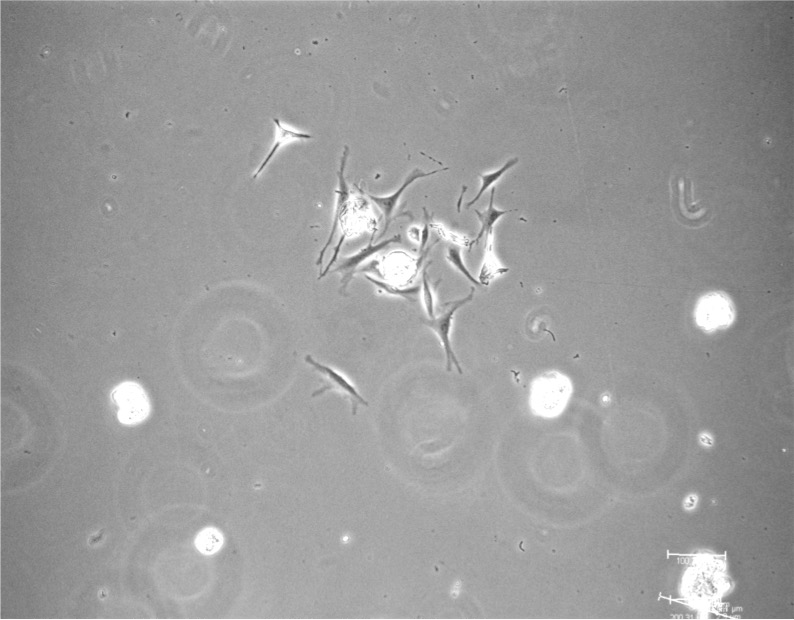 | 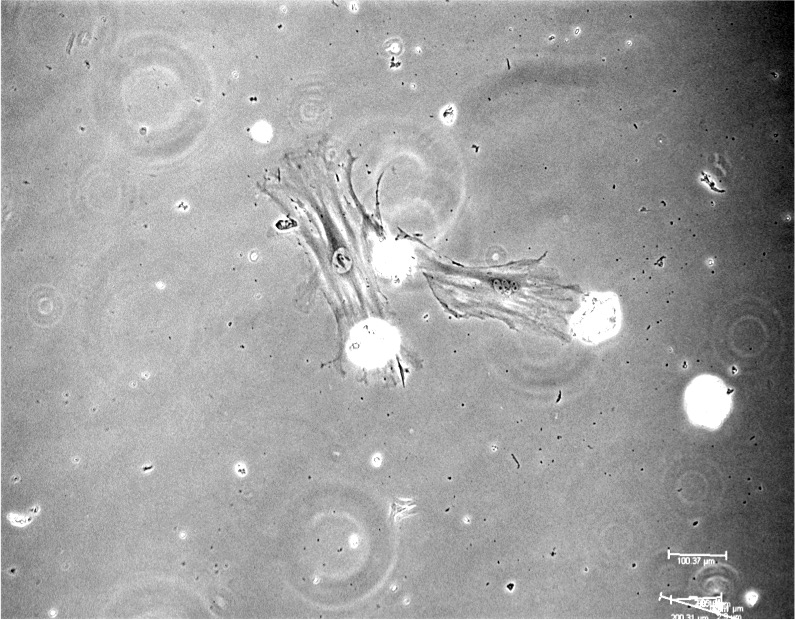 | 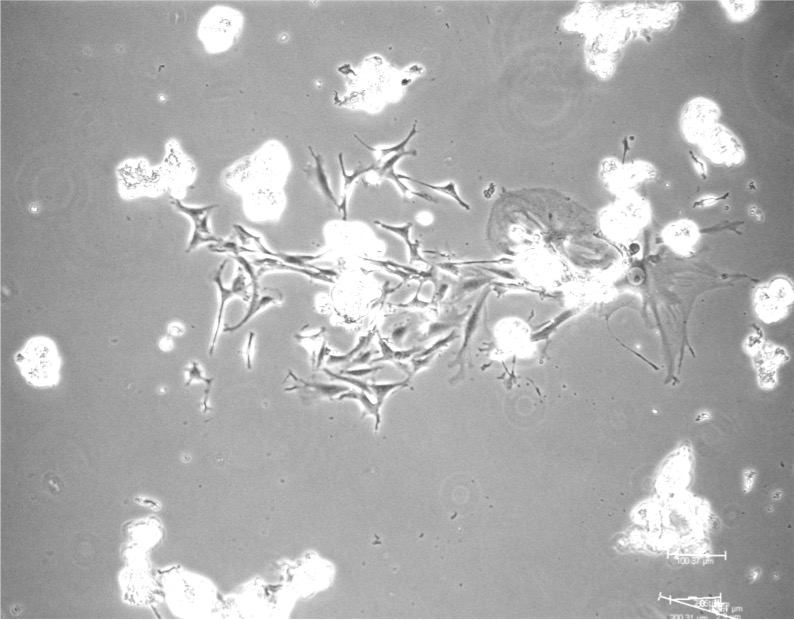 |

**Figure S7. Primary glomerular cell outgrowth colony at Day 5 in culture.** Live cell brightfield images of 12 separate patches of cells are shown. A) Every glomeruli shows cell outgrowth. WT glomerular cells that have moved out appear uniform in size. B) Numerous glomeruli from Alport KO mice have few outgrowths from glomeruli. Cells that have moved out from individual glomeruli are heterogeneous in size and morphology.

**Figure S8. Podocyte-specific WT-1 staining of reseeded glomerular outgrowth.** A. Podocytes stained for WT-1, Phalloidin, and DAPI are shown with merged image. B. Selected example of a low magnification image of primary podocytes identified by WT-1 positive staining, that was used to quantify that primary cells were >85% pure podocytes.

| **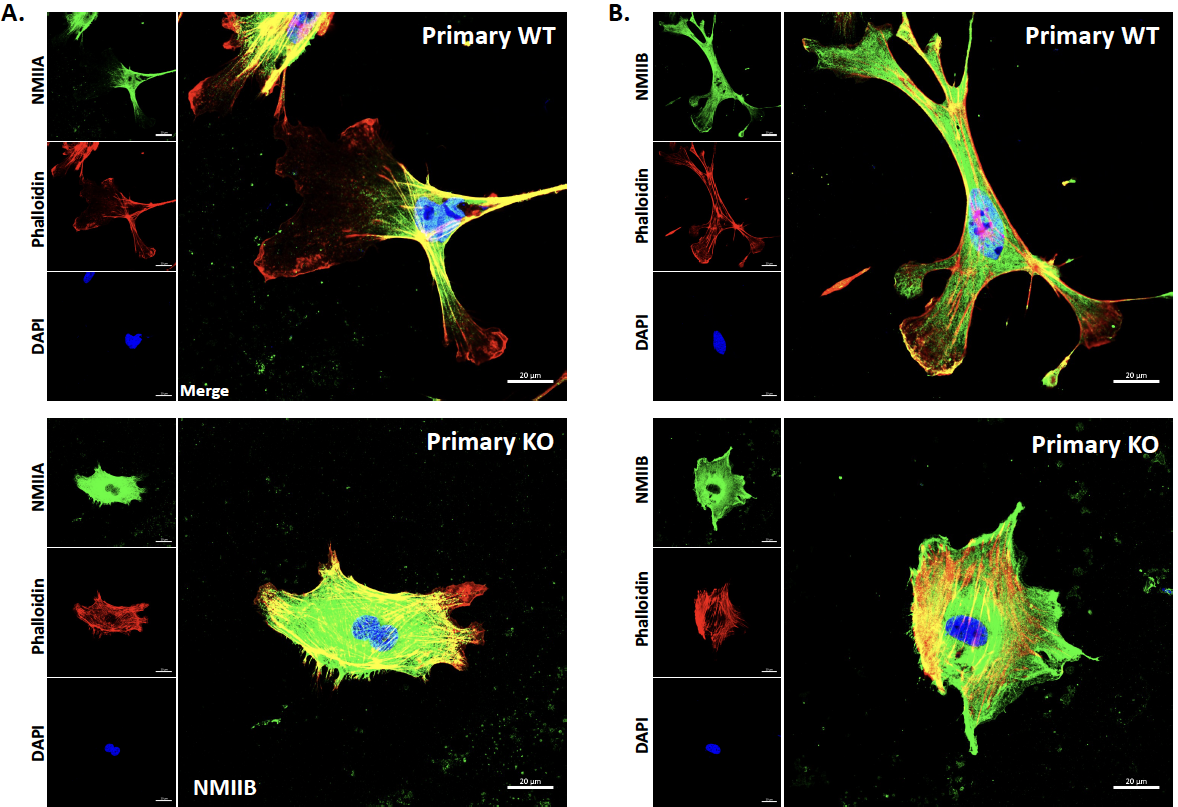** |
| --- |
| **Figure S9. Immunostaining of NMIIA/IIB in primary podocytes isolated from WT and Alport COL4α3 KO (KO) glomeruli.** Representative images of A) NMIIA and B) NMIIB co-stained with phalloidin to detect F-actin in primary podocytes from WT and KO mouse kidney. Left panels show the respective separated channel images for the enlarged merged image. NMIIA distribution is more centralized, and in both WT and KO cells, F-actin regions that are devoid of NMIIA are evident, whereas NMIIB distribution extends to F-actin positive edges of membrane extensions in both WT and KO podocytes. The scale bar represents 20 μm. |

**Figure S10. Comparison of WNK1 expression in podocyte cell lines and mouse primary podocytes.**

**Figure S11. Effects of WNK1 inhibition on NMIIB and vinculin distribution in isolated glomeruli. A) NMIIB localization shown in rendered 3D image of 9 slices (8um).** Arrows point to NMIIB-positive capillary loops where podocyte foot processes are located. B) Low magnification image of isolated glomeruli stained for vinculin after WNK1 inhibition *ex vivo*.


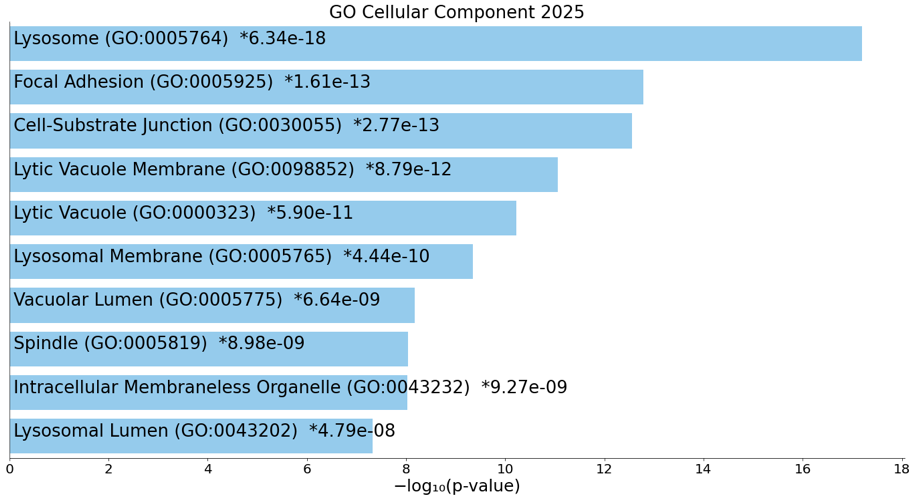


**Figure S12. Gene ontology analysis of differentially regulated genes in RNAseq comparison between WT and Alport KO primary podocytes, using Enrichr.** Total number of DEGs was 2758 after selection for genes with mean count >10, and -0.5>log2 fold change>0.5, N=1.
